# *Liriodendron* genome sheds light on angiosperm phylogeny and species–pair differentiation

**DOI:** 10.1038/s41477-018-0323-6

**Published:** 2018-12-17

**Authors:** Jinhui Chen, Zhaodong Hao, Xuanmin Guang, Chenxi Zhao, Pengkai Wang, Liangjiao Xue, Qihui Zhu, Linfeng Yang, Yu Sheng, Yanwei Zhou, Haibin Xu, Hongqing Xie, Xiaofei Long, Jin Zhang, Zhangrong Wang, Mingming Shi, Ye Lu, Siqin Liu, Lanhua Guan, Qianhua Zhu, Liming Yang, Song Ge, Tielong Cheng, Thomas Laux, Qiang Gao, Ye Peng, Na Liu, Sihai Yang, Jisen Shi

**Affiliations:** 1 Key Laboratory of Forest Genetics and Biotechnology, Ministry of Education of China, Co-Innovation Center for the Sustainable Forestry in Southern China, Nanjing Forestry University, Nanjing, China; 2 BGI Genomics, BGI-Shenzhen, Shenzhen, China; 3 Warnell School of Forestry and Natural Resources, University of Georgia, Athens, GA USA; 4 The Jackson Laboratory for Genomic Medicine, Farmington, CT USA; 5 College of Biology and the Environment, Nanjing Forestry University, Nanjing, China; 6 Department of Surgical and Radiological Sciences, Schools of Veterinary Medicine and Medicine, University of California, Davis, Davis, CA USA; 7 General Station of Forest Seedlings of Hubei Provincial Forestry Department, Wuhan, China; 8 Institute of Botany, Chinese Academy of Sciences, Beijing, China; 9 BIOSS Centre for Biological Signalling Studies, Faculty of Biology, Albert-Ludwigs-Universität Freiburg, Freiburg, Germany; 10 State Key Laboratory of Pharmaceutical Biotechnology, School of Life Sciences, Nanjing University, Nanjing, China

**Keywords:** Population genetics, Plant evolution, Next-generation sequencing, Genome evolution

## Abstract

The genus *Liriodendron* belongs to the family Magnoliaceae, which resides within the magnoliids, an early diverging lineage of the Mesangiospermae. However, the phylogenetic relationship of magnoliids with eudicots and monocots has not been conclusively resolved and thus remains to be determined^1–6^. *Liriodendron* is a relict lineage from the Tertiary with two distinct species—one East Asian (*L. chinense* (Hemsley) Sargent) and one eastern North American (*L. tulipifera* Linn)—identified as a vicariad species pair. However, the genetic divergence and evolutionary trajectories of these species remain to be elucidated at the whole-genome level^7^. Here, we report the first de novo genome assembly of a plant in the Magnoliaceae, *L. chinense*. Phylogenetic analyses suggest that magnoliids are sister to the clade consisting of eudicots and monocots, with rapid diversification occurring in the common ancestor of these three lineages. Analyses of population genetic structure indicate that *L. chinense* has diverged into two lineages—the eastern and western groups—in China. While *L. tulipifera* in North America is genetically positioned between the two *L. chinense* groups, it is closer to the eastern group. This result is consistent with phenotypic observations that suggest that the eastern and western groups of China may have diverged long ago, possibly before the intercontinental differentiation between *L. chinense* and *L. tulipifera*. Genetic diversity analyses show that *L. chinense* has tenfold higher genetic diversity than *L. tulipifera*, suggesting that the complicated regions comprising east–west-orientated mountains and the Yangtze river basin (especially near 30° N latitude) in East Asia offered more successful refugia than the south–north-orientated mountain valleys in eastern North America during the Quaternary glacial period.

## Abstract (web summary)

A high-quality reference genome for the plant *Liriodendron chinense* places magnoliids as sister to the clade consisting of eudicots and monocots. Population genomic analyses unravel the evolution of two distinct species in the genus *Liriodendron*.

## Main

The Magnoliaceae, a family in the order Magnoliales, is an early diverging lineage of the Mesangiospermae (core angiosperms)^8^, and thus, it possesses a crucial phylogenetic position for better understanding the evolution of the extant flowering plants. However, the relationships among magnoliids, eudicots, and monocots have not been conclusively resolved despite previous valuable attempts^2,5,6^. The *Liriodendron* genus, which belongs to the subfamily Liriodendroideae of the Magnoliaceae, consisted of several species distributed throughout the Northern Hemisphere until the Late Tertiary, but now comprises only of a pair of sister species with a classic intercontinental disjunction distribution: one in East Asia (*L. chinense*) and the other in eastern North America (*L. tulipifera*). These two Tertiary relict *Liriodendron* species have been suggested to have diverged during the middle to late Miocene^7,9^, a reflection of range restrictions resulting from extinctions in the late Cenozoic^10^. Moreover, this pair of species is a perfect verification of the second prediction of the geographic speciation theory, which was proposed to explain the origin of species^11,12^.

Here, we combined three different sequencing technologies (that is, short-read sequencing, long-read sequencing and optical mapping) to de novo assemble the *L. chinense* genome. First, we achieved ~327.11 gigabases (Gb) of clean Illumina paired-end reads (Supplementary Table 1), ~147.89 Gb of corrected PacBio long reads (length longer than 2 kilobases (kb); Supplementary Table 2) and ~315.41 Gb of Bionano genome map data (Supplementary Table 3). We estimated the genome size to be 1.75 Gb based on Illumina data (Supplementary Fig. 1 and Supplementary Table 4), which was consistent with the estimation of ~1.8 Gb using flow cytometry (Supplementary Note). Then, we assembled the genome of *Liriodendron* into 4,624 contigs with an N50 length of 1.43 megabases (Mb) using Falcon (Supplementary Table 5). Furthermore, this assembly of long reads was integrated with a Bionano optical map to create a hybrid assembly consisting of 3,711 scaffolds totalling 1.74 Gb with an N50 length of 3.53 Mb (Supplementary Table 5). Finally, we anchored 529 scaffolds totalling ~1.37 Gb to a genetic map with 19 linkage groups, using a total of 1,576 microsatellite markers (Supplementary Fig. 2 and Supplementary Table 6). A high-confidence set of 35,269 gene models was constructed using the genome annotation pipeline MAKER (Supplementary Fig. 3), with 83.59% of genes being assigned putative functional annotations (Supplementary Table 7). To assess the quality of the assembly, we compared ten bacterial artificial chromosomes (BACs), in which potential repeat regions were masked (Supplementary Note), with assembled scaffolds, resulting in an average coverage of 99.75% (Supplementary Fig. 4). Of all 66,934 unigenes (>200 base pairs (bp)) assembled de novo by RNA sequencing (RNA-Seq), more than 90% had a length coverage of greater than 90% within a single scaffold (Supplementary Table 8). In addition, 1,300 (90.28%) genes of the BUSCO plant set were covered by the *Liriodendron* genome (Supplementary Table 9).

**Fig. 1:**
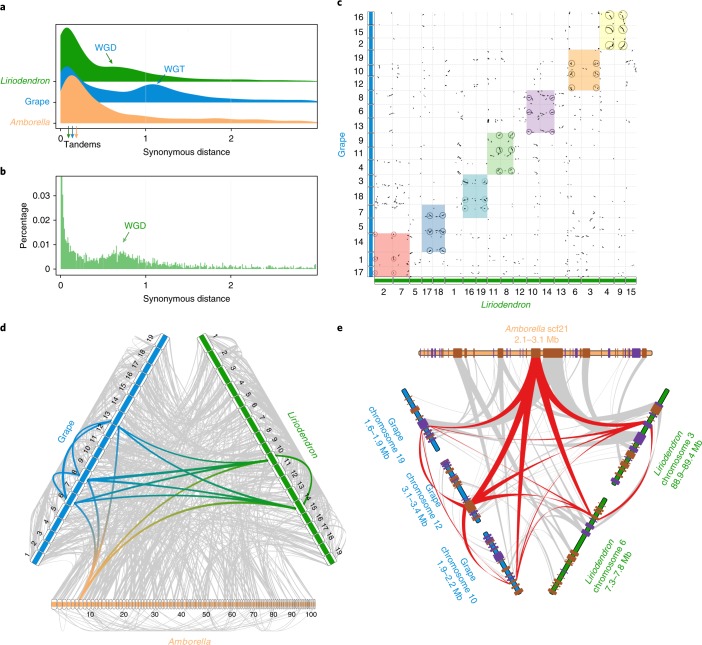
*Liriodendron* lineage-specific WGD. **a**, *K*_s_ distributions for the whole paranome identified from the whole genome of *Liriodendron* (green), grape (blue) and *Amborella* (orange). WGT, whole-genome triplication. **b**, *K*_s_ distribution for the whole paranome identified from the whole transcriptome of *L. chinense*. **c**, Comparison of *Liriodendron* and grape genomes. Dot plots of orthologues show a 2–3 chromosomal relationship between the *Liriodendron* genome and grape genome. **d**, Macrosynteny patterns show that a typical ancestral region in the basal angiosperm *Amborella* can be tracked to up to two regions in *Liriodendron* and to up to three regions in the grape. Grey wedges in the background highlight major syntenic blocks spanning more than 30 genes between the genomes (highlighted by one syntenic set shown in colour). **e**, Microcollinearity patterns between genomic regions from *Amborella*, *Liriodendron* and the grape. Rectangles represent predicted gene models, with purple and brown showing relative gene orientations. Grey wedges connect matching gene pairs, with two sets highlighted in red.

The genome size of *L. chinense* is larger than those of most sequenced angiosperms (Supplementary Fig. 5). We further investigated two pertinent aspects of genome evolution—whole-genome duplication (WGD) events and transposable element bursts—both of which have had profound effects on plant genome evolution^13^. The fraction of synonymous substitutions per synonymous site (*K*_s_) distributions of paralogues in the *Liriodendron* genome and transcriptome clearly illustrate the occurrence of a single WGD event experienced by *Liriodendron* (Fig. 1a,b). It has been firmly established that whole-genome triplication (mechanistically originating as two successive WGDs) occurred in the grape^14^, and there is no evidence for lineage-specific polyploidy events in *Amborella*^15^. By performing a comparative genomic analysis of *Vitis* with *Amborella* and *Liriodendron*, we identified 3:1 and 3:2 syntenic depth ratios in the *Vitis*–*Amborella* (Supplementary Fig. 6) and *Vitis–Liriodendron* (Fig. 1c and Supplementary Fig. 7) comparisons, respectively. Furthermore, we mapped the complete repertoire of 1–2–3 orthologous regions in the *Amborella–Liriodendron*–*Vitis* genome comparison (Fig. 1d,e). Thus, from these data, we conclude that a single *Liriodendron* lineage-specific WGD event occurred, consistent with the results of the fourfold synonymous third-codon transversion position analysis (Supplementary Fig. 8). We speculated that the *Liriodendron* WGD event occurred approximately 116 million years ago (Ma) with a synonymous substitution rate of 3.02 × 10^−9^ synonymous substitutions yr^−^^1^ (ref. ^16^). Considering the possibly overestimated synonymous substitution rate^16^ and the divergence time of 113–128 Ma between the families Magnoliaceae and Lauraceae^17^, the WGD detected in the *Liriodendron* genome probably predated the separation of these two families.

Transposable elements account for 61.64% of the *Liriodendron* genome (Supplementary Tables 10 and 11). Long terminal repeat (LTR) retrotransposons are the most abundant transposable element, representing 56.25% of the assembly (Supplementary Table 11). Among the LTR retrotransposons, *Gypsy* elements are much more abundant than *Copia* elements (Supplementary Table 12 and Supplementary Fig. 9). In addition, transposable elements are unevenly distributed across the *Liriodendron* genome and tend to accumulate in intergenic regions rather than genic regions and regions adjoining genes (Supplementary Fig. 10), probably as a result of natural selection due to the potential detrimental effects of transposable elements on gene expression^18^. With respect to the genic regions, transposable elements have an unequal distribution between exons and introns, and there is an obvious bias towards transposable element accumulation in introns compared with exons (Supplementary Fig. 11), consistent with the natural selection hypothesis, although introns may play an important role in gene expression^19^. Furthermore, long interspersed nuclear element-1 has an abnormally high rate of accumulation in genic regions, in contrast with the pattern shown by other transposable elements (Supplementary Fig. 12 and Supplementary Table 13). Moreover, we analysed the divergence time distribution for all LTRs in the *Liriodendron* genome and found a *K*_s_ peak at 0.05 (Supplementary Fig. 13). We assumed an intergenic nucleotide substitution rate of 1.51 × 10^−9^ that was roughly twice as low as that within the genic regions (Supplementary Note), resulting in an insertion time of ~16 Ma. Overall, these results show that an ancient WGD event that occurred approximately 116 Ma, followed by a more recent burst of transposable element insertion that occurred approximately 16 Ma, have both contributed to the expansion of the *Liriodendron* genome.

**Fig. 2:**
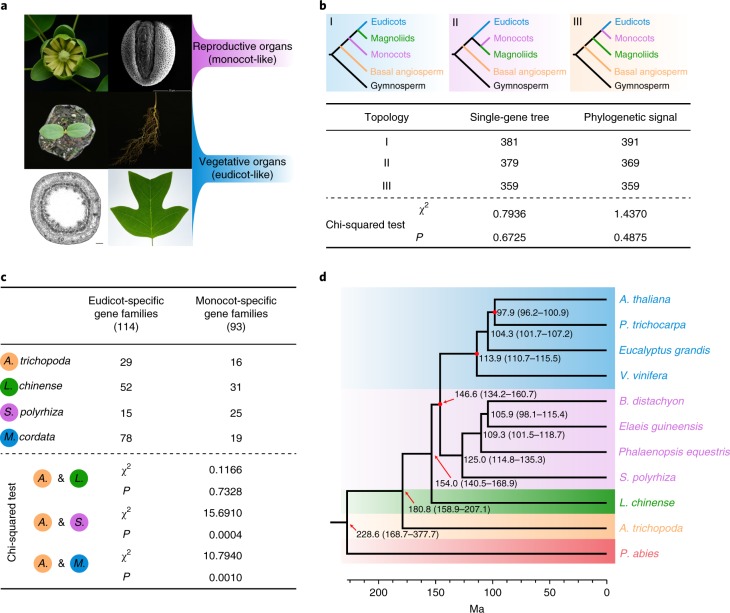
Phylogenetic relationships among magnoliids, eudicots and monocots. **a**, *Liriodendron* shows typical features of monocots in its reproductive organs (flower parts in multiples of three and monosulcate pollen grains) and of eudicots in its vegetative organs (two cotyledons, a taproot system, a eudicot-like stem cross-section and netted venation). These experiments were repeated independently at least ten times with similar results. Scale bar, 200 µm. **b**, Three topologies that coincided with three alternative phylogenetic hypotheses are plotted, and the results of a chi-squared test of the orthogroup numbers supporting each topology are shown below, revealing no statistically significant difference in topology preference. **c**, The eudicot- and monocot-specific gene families present in *Liriodendron* are statistically similar to those present in *Amborella*, whereas *Spirodela polyrhiza* has a bias towards monocot-specific gene families, and *Macleaya cordata* has a bias towards eudicot-specific gene families when compared with *Amborella*. **d**, Dated phylogeny for 11 plant species with *Picea abies* as an outgroup. A time scale is shown at the bottom, and red points in some nodes indicate fossil calibration points.

Some features of the *Liriodendron* phenotype are typical of both monocots and eudicots (Fig. 2a), which is consistent with the obscure phylogenetic relationships among magnoliids, monocots and eudicots. To investigate which of the three previously proposed tree topologies is most likely to be true (that is: (1) ((monocots, (eudicots, magnoliids)), basal angiosperm); (2) ((eudicots, (monocots, magnoliids)), basal angiosperm); or (3) ((magnoliids, (monocots, eudicots)), basal angiosperm) (Supplementary Table 14)), we selected an additional six eudicots, six monocots, three magnoliids and one basal angiosperm, with one gymnosperm being the outgroup (Supplementary Fig. 14), to construct individual orthogroups. In this way, we could use as many gene families as possible to depict a broad picture of the phylogeny. After careful evaluation and selection (Supplementary Note), we finally obtained 502 low-copy orthogroups, with 172 orthogroups (34.26%) supporting topology I, 155 orthogroups (30.88%) supporting topology II and the final 175 orthogroups (34.86%) supporting topology III (Fig. 2b), with no statistically significant difference among the three topologies (*χ*^2^ = 1.3904; *P* = 0.4990). Based on these 502 low-copy orthogroups, quantification of differences in gene-wise log-likelihood scores (ΔGLS) among these three alternative topologies^20^ showed an equal distribution of phylogenetic signals for each topology at the gene level (Supplementary Fig. 16). Further excluding orthogroups whose ΔGLS values were outliers (Supplementary Note), we obtained 481 low-copy orthogroups, with a lack of statistical significance among the orthogroups supporting each of the three alternative topologies (Fig. 2b; *χ*^2^ = 0.2162; *P* = 0.8975). These results explain why all three possible topologies have been observed in previous studies using different datasets (Supplementary Table 14) and suggest that rapid diversification occurred in the common ancestor of magnoliids, eudicots and monocots, which might be responsible for the phylogenetic incongruence in previous studies.

To further confirm the *Liriodendron* phylogeny, a coalescent-based species tree was constructed using the 502-orthogroup dataset, and this tree supported topology III with low bootstrap support (Supplementary Fig. 17a). Additionally, we performed coalescent-based species tree construction based on the 481-orthogroup dataset, yielding a topology identical to topology III with a bootstrap value increasing from 50 to 54% (Supplementary Fig. 17b). Furthermore, we performed a phylogenetic analysis on the basis of a concatenated sequence alignment of 78 chloroplast genes, yielding a topology consistent with topology III with strong bootstrap support (Supplementary Fig. 18). To continue our investigation, we identified both eudicot- and monocot-specific gene families present in the *Liriodendron* genome based on the PLAZA 3.0 Monocots database (Supplementary Fig. 19). The gene families from either clade were not significantly over-represented in *Liriodendron* compared with *Amborella* (*χ*^2^ = 0.1166; *P* = 0.7328), whereas a monocot plant and a eudicot plant both showed significant biases towards their respective gene families (Fig. 2c). Overall, considering our results, including the mosaic phenotypic characterization, individual and multiple gene tree reconstructions, and lineage-specific gene family identification, we suggest a topology in which eudicots and monocots form a clade that is sister to magnoliids, represented by *Liriodendron*, with the basal angiosperm *Amborella* being the next group (Fig. 2d); that is, magnoliids arose before the divergence of eudicots and monocots. Thus, the phylogenetic analysis incorporating the *Liriodendron* genome provides additional insights into the systematic position and evolution of magnoliids.

**Fig. 3:**
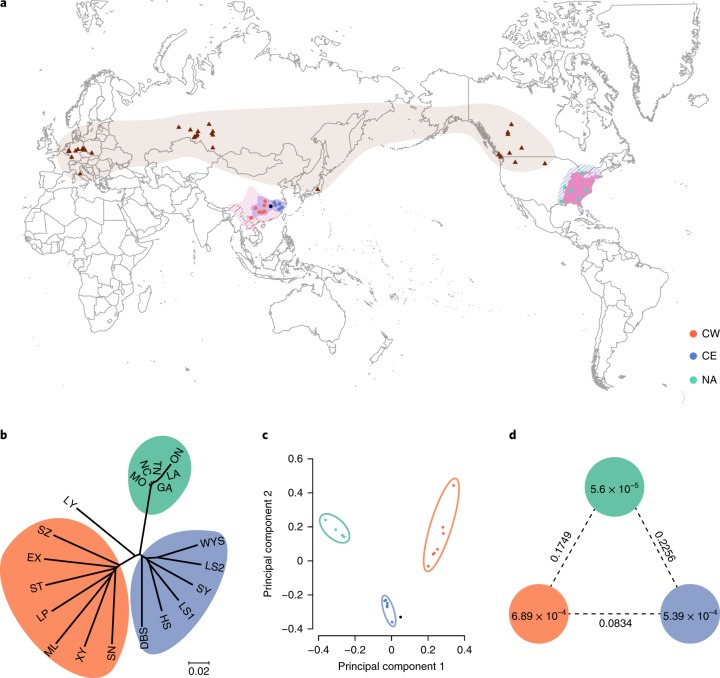
Geographic distribution and population diversity of *Liriodendron* accessions. **a**, Geographic distribution of *Liriodendron* accessions. Brown triangles represent the fossil distribution of *Liriodendron* plants in high-latitude regions of the Northern Hemisphere. Fringe patterns show two principal refugia where Tertiary relict floras occurred: southern East Asia and eastern North America. The natural distributions of *L. chinense* and *L. tulipifera* are plotted, with coloured dots representing individual *Liriodendron* accessions. **b**, Neighbour-joining tree of all accessions constructed from whole-genome SNPs. Accessions coming from the same geographic areas are grouped together and coloured corresponding to the colours used in **a**. LY, Liu Yang; SZ, Sang Zhi; EX, E Xi; ST, Song Tao; LP, Li Ping; ML, Meng La; XY, Xu Yong; SN, Sui Ning; DBS, DaBie Shan; HS, Huang Shan; LS1, Lushan_1; SY, Song Yang; LS2, Lushan_2; WYS, WuYi Shan; ON, Ontario; LA, Louisiana; GA, Georgia; TN, Tennessee; NC, North Carolina; MO, Missouri. **c**, Principal component analysis plots of the first two components for all 20 accessions, with dots coloured corresponding to their provenances. **d**, Nucleotide diversity (*π*) and population divergence (*F*_ST_) across the three groups. The value in each circle represents a measure of nucleotide diversity for this group, and the value on each line indicates the population divergence between the two groups.

At present, the *Liriodendron* genus contains only two species in regions with a humid subtropical climate, and has partially expanded to the southern margin of the warm temperate climate zone of the Northern Hemisphere^21,22^ (Fig. 3a and Supplementary Fig. 20). However, a number of extinct *Liriodendron* species were once widely distributed in relatively high-latitude regions of the Northern Hemisphere before a general cooling of the climate occurred during in the Late Tertiary^23^, based on fossil records of seeds and leaves (Fig. 3a and Supplementary Fig. 21). To explore the historical demographic fluctuations and present-day genetic diversity within these two *Liriodendron* species, we resequenced 20 *Liriodendron* accessions, including 14 *L. chinense* individuals and six *L. tulipifera* individuals (Fig. 3a, Supplementary Fig. 22 and Supplementary Table 15).

On the basis of phylogenetic analysis of a whole-genome single nucleotide polymorphism (SNP) analysis, we found that these *Liriodendron* accessions formed three distinct phylogenetic groups (Fig. 3b and Supplementary Fig. 23). This was further supported by a principal component analysis (Fig. 3c) and structure analysis (Supplementary Fig. 24). All *L. chinense* individuals from western China (CW) clustered together, and the rest of the *L. chinense*, collected from eastern China (CE), clustered into the second group. The third group comprised all *L. tulipifera* individuals collected from North America (NA). It is evident that the NA group is phylogenetically positioned between the two *L. chinense* groups and more closely related to the CE group, suggesting that the earliest divergence occurred between the populations in eastern China and those in western China, followed by differentiation between the eastern Chinese populations and North American populations. This pattern is supported by the phenotypic analysis, which shows that all three groups share one leaf morphological feature, while the CE and NA groups have their own unique leaf morphological feature (Supplementary Fig. 25). Fossil records indicate that similar leaf morphological features to those in the western and eastern China groups had already emerged in two extinct *Liriodendron* species^24,25^, again suggesting that these two *L. chinense* groups may have diverged a very long time ago, possibly preceding the intercontinental differentiation between *L. chinense* and *L. tulipifera* (Supplementary Fig. 25).

**Fig. 4:**
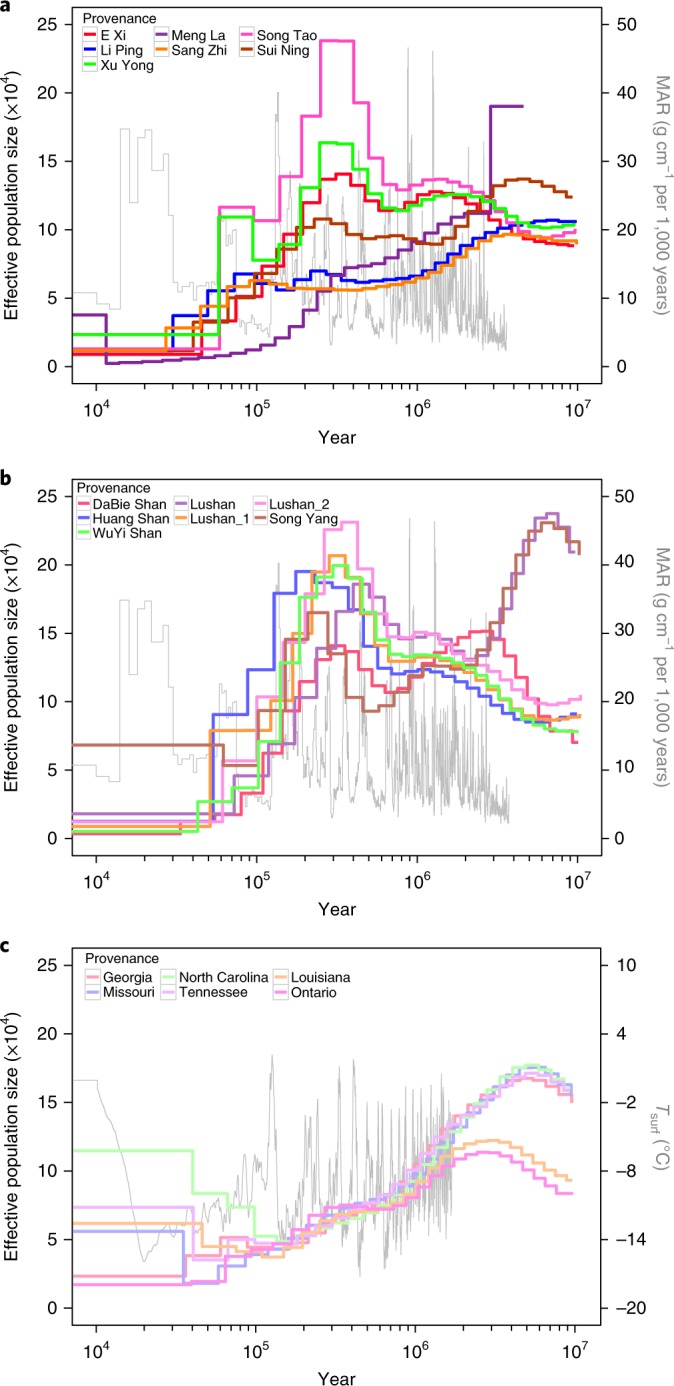
Historical fluctuations in effective population size. **a**–**c**, Plots of PSMC results for 20 individuals (7 from western China (**a**); 7 from eastern China (**b**); and 6 from North America (**c**)), as indicated in each legend. The grey lines represent the mass accumulation rate (MAR) of the Chinese Loess Plateau in **a** and **b**, and the atmospheric surface air temperature relative to the present in **c**.

Nucleotide diversity (*π*) analysis shows that the CW group has the highest genetic diversity, followed by the CE group, and that the genetic diversity of the NA group is tenfold lower than that of the CW group (Fig. 3d). An analysis of demographic history using the pairwise sequentially Markovian coalescent (PSMC) model^26^ shows that the two groups from China both had population size peaks at approximately 0.4 Ma and declined afterwards, whereas the NA group population size peak occurred much earlier and continuously declined since approximately 2.3 Ma (Fig. 4), indicating that the populations in eastern China and those in western China underwent a similar demographic history different from that in North American populations. We also calculated genetic differentiation statistics (fixation index; *F*_ST_) among the three *Liriodendron* groups, indicating that the genetic differentiation (*F*_ST_ = 0.2055) between the NA group and the CW group was slightly lower than that (*F*_ST_ = 0.2707) between the NA group and the CE group (Fig. 3d). In addition, we also found that the CW group had the highest level of individual differences compared with the other two geographical groups (Supplementary Fig. 26).

The natural distribution areas of these two *Liriodendron* species on their respective continents are highly consistent with the two principal areas where Tertiary relict floras occur^23^ (Fig. 3a). Although *Liriodendron* species were once distributed over the high-latitude regions of Europe (Fig. 3a), the east–west-orientated mountains are thought to have blocked their southward migration during global cooling in the Late Tertiary and subsequent Quaternary glaciations^27^, finally leading to the extinction of *Liriodendron* in Europe^7^. With respect to the *Liriodendron* that survived in East Asia and eastern North America, the higher genetic diversity of *L. chinense* compared with *L. tulipifera* is consistent with the greater number of suitable refugia in East Asia^28,29^. In this study, we observed a sustained population decrease during the whole Quaternary glaciation in all *L. tulipifera* accessions and a population recovery approximately 0.3–0.4 Ma in all *L. chinense* accessions (Fig. 4), which may have contributed considerably to the severe loss of genetic diversity in *L. tulipifera* and the relatively high retention of genetic diversity in *L. chinense* (Fig. 3d and Supplementary Fig. 27), respectively. The population recovery observed in all *L. chinense* accessions occurred in the interglacial stage between the Guxiang Glaciation (0.3–0.13 Ma) and Naynayxungla Glaciation (0.72–0.5 Ma)^30^. Considering that the Naynayxungla Glaciation was the most extensive glaciation, including large ice caps and massive valley glaciers, and the following Guxiang Glaciation was characterized by valley glaciers only^30^, we speculate that the temperature recovery and deglaciation during this interglacial stage provided a foundation for *L. chinense* population recovery within East Asian refugia. Consequently, in addition to the higher habitat diversity within East Asian refugia^29^, a suitable living environment during the interglacial stage between the Naynayxungla and Guxiang glaciations may have contributed to the retention, restoration and augmentation of *L. chinense* genetic diversity.

## Methods

### Plant materials and sequencing

For genome sequencing, we collected fresh leaves from an adult plant of *L. chinense* grown in Lushan, which is located in the Jiangxi province of China. For Illumina sequencing, four series of paired-end sequencing libraries with insert sizes of 170, 250, 500 and 800 bp were constructed and subsequently sequenced on the Illumina HiSeq 2000 platform, ultimately resulting in 327.11 Gb clean reads. For PacBio single-molecule real-time sequencing, sequencing libraries with 20-kb DNA inserts were constructed and subsequently sequenced on the Pacific Biosciences RSII instrument, ultimately resulting in a total of 150.18 Gb subread with an N50 length of 15.96 kb for the genome assembly. In addition, purified DNA was labelled at Nt.BspQI sites using the IrysPrep kit, and a 315.41 Gb optical map of the sample was produced from the BioNano Irys system. In addition, abundances of 17-nucleotide k-mers from 170- and 250-bp Illumina sequencing libraries were used to estimate the genome size.

### De novo assembly

The *Liriodendron* genome was de novo assembled using FALCON (https://github.com/PacificBiosciences/FALCON) based on PacBio long reads (only reads longer than 10 kb were used in the assembly). Errors in the PacBio reads were corrected within the FALCON pipeline. Contigs was first polished based on raw PacBio data and finally corrected using Illumina short reads with Pilon^31^. A hybrid assembly was created based on contigs and optical maps using the Bionano Solve Pipeline (https://bionanogenomics.com/support-page/bionano-access/). Then, the corrected PacBio long reads were used for superscaffold gap filling using PBJelly^32^. We constructed a reference genetic map of *L. chinense* based on an F_1_ population of 150 plants from a cross between *L. chinense* and *L. tulipifera* using JoinMap 4.0 (ref. ^33^). Markers with inconsistent placement were manually screened and the collinearity of common markers was inspected using MapChart 2.2 (ref. ^33^). Markers in common were used as anchor points. Possible chimeric scaffolds were identified as those containing sequences of markers mapped to different locations in the same linkage group or different linkage groups, and these scaffolds were manually inspected. This process generated 19 *Liriodendron* pseudomolecules.

### Genome assessment

We assessed the coverage of the genome assembly by mapping 89 BACs back to assembly with 97% of these BAC sequences covered without any obvious misassemblies. A comparison of 9 randomly chosen BACs sequenced by 454 sequencing technology indicated a low error rate. In addition, we used the BUSCO^34^ database to assess the genome assembly. We also validated the assembled genome using 66,934 unigenes (length ≥ 200 bp) from RNA-Seq.

### Repeat annotation

We identified tandem repeats and transposable elements separately. Tandem repeats were predicted using Tandem Repeats Finder 4.04 (ref. ^35^). For transposable element identification, we performed a combination of similarity-based and de novo approaches. First, we used RepeatMasker with the Repbase 16.10 (ref. ^36^) database of known repeat sequences to search for transposable elements in the genome, and we additionally used RepeatProteinMask, implemented in RepeatMasker, to identify transposable elements by aligning the genome sequence to the transposable element protein database. Then, to apply our de novo approach, we constructed a repeat library generated by RepeatModeler^37^ with default parameters and ran RepeatMasker on the genome sequences, using the RepeatModeler consensus sequence as a library. Finally, all the repeat sequences identified by the different methods were combined into the final repeat annotation.

### Gene prediction

Gene model prediction was conducted by the MAKER pipeline^38^, integrating ab initio prediction with de novo assembled transcripts from short-read messenger RNA sequencing, isoform-sequencing full-length transcripts, and protein homology data. A high-confidence gene model was constructed by further removing transposons and low-confidence predictions. Gene functional annotation was performed using the Swiss-Prot and TrEMBL databases^39^, while motifs and domains were annotated using InterProScan^40^ by searching against publicly available protein databases. Descriptions of gene products (that is, Gene Ontology terms) were retrieved from the corresponding InterPro entries. We also mapped the *Liriodendron* reference genes to KEGG^41^ pathway maps.

Transfer RNA genes were predicted based on tRNAscan-SE^42^. Ribosomal RNA fragments were identified by aligning plant ribosomal RNA sequences^43^ to the *Liriodendron* genome by BLASTN^44^. micro RNA and small nuclear RNA genes were detected by INFERNAL^45^ software against the Rfam database^46^ (release 9.1).

### Genome synteny

We performed synteny searches to compare the *L. chinense* genome structure with that of the grape and *Amborella* genomes using MCscan^47^, requiring at least five gene pairs per syntenic block. The resulting dot plots were inspected to confirm the paleoploidy level of *L. chinense* in relation to the other genomes by counting the syntenic depth in each genomic region.

*K*_s_ values for homologous gene pairs were calculated as described in Maere et al.^48^. Fourfold synonymous third-codon transversion position values were calculated for syntenic segments from the concatenated alignments and constructed by dividing the number of transversions at all fourfold degenerate third-codon positions by the number of fourfold degenerate third-codon positions.

### Phylogenetic analysis

Orthogroups were constructed with 14 other sequenced plants—6 eudicots (*Arabidopsis thaliana*, *Populus trichocarpa*, *Vitis vinifera*, *Coffea canephora*, *Ipomoea nil* and *Fraxinus excelsior*); 6 monocots (*Brachypodium distachyon*, *Xerophyta viscosa*, *Asparagus officinalis*, *Musa acuminata*, *Ananas comosus* and *Oryza sativa*); 1 basal angiosperm (*Amborella trichopoda*); and 1 gymnosperm (*Gnetum montanum*)—and three other magnoliid transcriptome datasets, including two sequenced in this study (*Magnolia grandiflora* and *Michelia alba*) and one available in Ibarra-Laclette et al.^49^ (*Persea americana*), using the software OrthoFinder^50^. We selected low-copy orthogroups with the number of putative orthologues less than two in each species, and putative orthologues were found in at least four eudicots, four monocots, three magnoliids, one basal angiosperm and one gymnosperm, resulting in 1,163 orthogroups. Then, each orthogroup was aligned using Clustal Omega^51^, and all alignments were further trimmed using TrimAl 1.2 (ref. ^52^). Next, we constructed 1,163 single-gene trees using RAxML^53^ with the PROTCATWAG mode. Then, we compared these single-gene trees with the species tree and screened them as described in Zeng et al.^6^. Finally, after careful examination, a total of 502 low-copy orthogroups were selected for further analysis.

We also calculated the phylogenetic signal based on three alternative topological hypotheses and quantified the difference in gene-wise log-likelihood scores (ΔGLS) among each of the three topologies using RAxML^20,53^. To diminish the influence of tiny amounts of data on phylogenetic inference, we further excluded orthogroups with outlier ΔGLS values, defined as described in Shen et al.^20^. To estimate the species tree, we performed a coalescent-based approach using Astral 5.6.1 (ref. ^54^). We also performed phylogenetic analyses based on 78 chloroplast genes among 24 land plant species using RAxML^53^.

To estimate divergence time, we used PAML MCMCTREE^55^ to perform Bayesian estimation with soft fossil constraints^56^ based on 235 single-copy orthologous genes that are shared by *L. chinense* and 10 other species. Markov chain Monte Carlo analysis was run to sample 1,000,000 times with a sampling frequency of 50 and a burn-in of 5,000,000 iterations. We also used CAFE^57^ to identify gene families that had undergone expansions or contractions across the maximum likelihood tree.

### Resequencing and diversity analysis

DNA from 14 *L. chinense* and 6 *L. tulipifera* adult plants was extracted, and paired-end libraries with insert sizes of 100–150 bp were sequenced using Illumina technology at BGI. We first called SNPs using BWA^58^, GATK^59^ and SAMtools^60^, then annotated these SNPs using SNPEFF^61^, ultimately summarizing them by a customized Perl script.

The neighbour-joining phylogenetic tree was constructed using TreeBeST^62^ based on SNPs. Population structure and ancestry information was inferred using FRAPPE^63^ with the best *K* value determined by ADMIXTURE^64^ based on a cross-validation test. We additionally performed a principal component analysis using the STRATPCA programme from EIGENSOFT 3.2 (ref. ^65^).

Population genetic parameters, including nucleotide diversity (*π*)^66^ and the Watterson estimator (*θ*_w_)^67^, were estimated on the basis of the genotypes of each line at the SNP positions using BioPerl.

The PSMC model, which was originally applied to human genomes^26^ and subsequently also applied to plant genomes^15,68^, was applied to study the effective population sizes (*N*_e_) of the two *Liriodendron* species over time.

See the Supplementary Note for additional details.

### Reporting Summary

Further information on research design is available in the Nature Research Reporting Summary linked to this article.

## Supplementary information

Supplementary Information Supplementary Note, Supplementary Tables 1–15 and Supplementary Figures 1–27.

Reporting Summary

## Data Availability

The custom Perl script used for summarization of SNP annotation is available from the corresponding author upon request.

## Data availability

The raw reads and genome assembly have been deposited as a BioProject under accession PRJNA418360. The resequencing data for 20 *Liriodendron* individuals have been deposited as a BioProject under accession PRJNA418361.
