# Author Correction: *Liriodendron* genome sheds light on angiosperm phylogeny and species–pair differentiation

**DOI:** 10.1038/s41477-019-0368-1

**Published:** 2019-01-23

**Authors:** Jinhui Chen, Zhaodong Hao, Xuanmin Guang, Chenxi Zhao, Pengkai Wang, Liangjiao Xue, Qihui Zhu, Linfeng Yang, Yu Sheng, Yanwei Zhou, Haibin Xu, Hongqing Xie, Xiaofei Long, Jin Zhang, Zhangrong Wang, Mingming Shi, Ye Lu, Siqin Liu, Lanhua Guan, Qianhua Zhu, Liming Yang, Song Ge, Tielong Cheng, Thomas Laux, Qiang Gao, Ye Peng, Na Liu, Sihai Yang, Jisen Shi

**Affiliations:** 1 Key Laboratory of Forest Genetics and Biotechnology, Ministry of Education of China, Co-Innovation Center for the Sustainable Forestry in Southern China, Nanjing Forestry University, Nanjing, China; 2 BGI Genomics, BGI-Shenzhen, Shenzhen, China; 3 Warnell School of Forestry and Natural Resources, University of Georgia, Athens, GA USA; 4 The Jackson Laboratory for Genomic Medicine, Farmington, CT USA; 5 College of Biology and the Environment, Nanjing Forestry University, Nanjing, China; 6 Department of Surgical and Radiological Sciences, Schools of Veterinary Medicine and Medicine, University of California, Davis, Davis, CA USA; 7 General Station of Forest Seedlings of Hubei Provincial Forestry Department, Wuhan, China; 8 Institute of Botany, Chinese Academy of Sciences, Beijing, China; 9 BIOSS Centre for Biological Signalling Studies, Faculty of Biology, Albert-Ludwigs-Universität Freiburg, Freiburg, Germany; 10 State Key Laboratory of Pharmaceutical Biotechnology, School of Life Sciences, Nanjing University, Nanjing, China

**Keywords:** Population genetics, Plant evolution, Next-generation sequencing, Genome evolution

Correction to: *Nature Plants* 10.1038/s41477-018-0323-6, published online 17 December 2018.

In the Supplementary Information file originally published with this Letter, the authors mistakenly omitted Supplementary Table 14; this has now been amended.

