# Supplementary material for: Liriodendron genome sheds light on angiosperm phylogeny and species–pair differentiation
Source: Nat Plants. 2018 Dec 17;5(1):18–25. doi: 10.1038/s41477-018-0323-6 (PMC6784881; doi:10.1038/s41477-018-0323-6)
Supplement: Supplementary file 1 — Supplementary Note, Supplementary Tables 1–15 and Supplementary Figures 1–27. [file 41477_2018_323_MOESM1_ESM.pdf]

SUPPLEMENTARY INFORMATION
https://doi.org/10.1038/s41477-018-0323-6

nature plants

In the format provided by the authors and unedited.

Liriodendron genome sheds light on angiosperm phylogeny and species-pair differentiation

Jinhui Chen [1,11*], Zhaodong Hao [1,11], Xuanmin Guang [2,11], Chenxi Zhao [2,11], Pengkai Wang [1], Liangjiao Xue [1,3], Qihui Zhu [4], Linfeng Yang [2], Yu Sheng [1], Yanwei Zhou [1], Haibin Xu [5], Hongqing Xie [2], Xiaofei Long [1], Jin Zhang [6], Zhangrong Wang [1], Mingming Shi [2], Ye Lu [1], Siqin Liu [1], Lanhua Guan [7], Qianhua Zhu [2], Liming Yang [5], Song Ge [8], Tielong Cheng [5], Thomas Laux [9], Qiang Gao [2], Ye Peng [5], Na Liu [2*], Sihai Yang [10*] and Jisen Shi [1*]

[1]Key Laboratory of Forest Genetics and Biotechnology, Ministry of Education of China, Co-Innovation Center for the Sustainable Forestry in Southern China, Nanjing Forestry University, Nanjing, China. [2]BGI Genomics, BGI-Shenzhen, Shenzhen, China. [3]Warnell School of Forestry and Natural Resources, University of Georgia, Athens, GA, USA. [4]The Jackson Laboratory for Genomic Medicine, Farmington, CT, USA. [5]College of Biology and the Environment, Nanjing Forestry University, Nanjing, China. [6]Department of Surgical and Radiological Sciences, Schools of Veterinary Medicine and Medicine, University of California, Davis, Davis, CA, USA. [7]General Station of Forest Seedlings of Hubei Provincial Forestry Department, Wuhan, China. [8]Institute of Botany, Chinese Academy of Sciences, Beijing, China. [9]BIOSS Centre for Biological Signalling Studies, Faculty of Biology, Albert-Ludwigs-Universität Freiburg, Freiburg, Germany. [10]State Key Laboratory of Pharmaceutical Biotechnology, School of Life Sciences, Nanjing University, Nanjing, China. [11]These authors contributed equally: Jinhui Chen, Zhaodong Hao, Xuanmin Guang, Chenxi Zhao. *e-mail: chenjh@njfu.edu.cn; naliu@bgi.com; sihaiyang@nju.edu.cn; jshi@njfu.edu.cn

Supplementary Note

1. Genome sequencing and assembly

1.1 Plant materials and DNA preparation

An adult plant *L. chinense* grown in Lushan located in Jiangxi province of China was used for genome sequencing. For Illumina sequencing, fresh leaves were harvested and frozen immediately in liquid nitrogen for extracting genomic DNA by using a modified CTAB protocol[1]. We ran a DNA quality check on gel electrophoresis using agarose gels (0.3% agarose) for 24h at 30V. In addition, DNA purity was verified by NanoDrop™ Spectrophotometers ND-2000 (Thermo Fisher Scientific, Waltham, MA, USA). For Pacbio sequencing, DNA was extracted following the Mayjonade pipeline[2].

1.2 Whole genome sequencing

Whole genome sequencing for the *L. chinense de novo* genome was generated at Beijing Genome Institute, Shenzhen (BGI-Shenzhen, China). For Illumina sequencing, four paired-end libraries with insert sizes of 170, 250, 500 and 800 bp were constructed and sequenced (Supplementary Table 1). All libraries were constructed according to the manufacturer's instructions (Illumina). The quality of each library was validated using Qubit®, AGE. A total of 367.41 Gb raw data were generated using Illumina platforms, i.e., HiSeq 2000 (Supplementary Table 1). In addition, the *Liriodendron* genome was sequenced using 33 SMRT Cells with P6/C4 chemistry, resulting in a total of 147.89 Gb raw data with minimum subread length > 2kb (Supplementary Table 2). And, we also generated a total of 315.41Gb Bionano optical maps for further improvement of

the contiguity of the *Liriodendron* genome assembly (Supplementary Table 3). All sequence data have been deposited in the NCBI Sequence Read Archive under project PRJNA418360.

1.3 Raw data processing in Illumina data

Low quality reads were filtered out and potential sequencing errors were removed or corrected by *k*-mer frequency methodology. The following filtering criteria were applied to reduce effects of sequencing errors on the assembly, thereby ensuring high quality reads.

1) Reads with ambiguous bases (represented by the letter N) or poly-A structures.
2) Reads with ≥40% low-quality bases (base quality ≤7) in small insert size libraries (170, 250, 500, and 800 bp).
3) Reads with adapter contamination: reads with ≥10 bp aligned to the adapter sequence (≤3 bp mismatch allowed) were filtered out.
4) Small insert size reads in which read1 and read2 overlapped by ≥10 bp (10% mismatch allowed).
5) PCR duplications (reads were considered duplicates when read1 and read2 of the two paired-end reads were identical).

Low quality and duplicated reads were filtered out, 327.11 Gb of the *L. chinense* genome was retained for the coming assembly (Supplementary Table 1).

1.4 Genome size and heterozygosity estimation

A *k*-mer refers to an artificial sequence division of K nucleotides iteratively from sequencing reads. A raw sequence read with L bp contains (L – K + 1) *k*-mers, if the length of each *k*-mer is K bp. The frequency of each *k*-mer can be calculated from genome sequence reads. Frequencies of a *k*-mer along the sequence depth gradient follow a Poisson distribution in a given dataset, except for a higher representation of low frequencies due to sequencing errors, as sequencing errors affect the number of *k*-mers that may be orphan among all splitting *k*-mers. The genome size (G) is defined as G = K_num/K_depth, where the K_num is the total number of *k*-mers, and K_depth is the frequency occurring more frequently than other frequencies. In this research, we used K = 17 to estimate genomes size and a K_num value of 4,210,050,595. By plotting the occurrence of *k*-mers against the percentage of corresponding *k*-mers, we found that the peak depth was 24. Our results suggested that the *L. chinense* genome was approximately 1,750 Mbp (Supplementary Table 4).

In addition to the primary peak observed from the distribution of *k*-mer occurrence, we also noted that there was a secondary peak at approximately half of the major depth. This secondary peak reflected heterozygous regions of the *Liriodendron* genome, since *k*-mers of two separate alleles in heterozygous regions are not identical. As a consequence, *k*-mers mapping to the secondary peak are expected to have just half of the average sequencing depth of the primary peak. This secondary peak corresponds to a peak depth of 12 and simulated results show a 1.3% heterozygosity (Supplementary Fig. 1).

1.5 Genome size estimation using flow cytometry

For genome size estimation using flow cytometry, ‘Two-step’ Method with ‘Cystain PI absolute P’ buffer from sysmex Partec (art. Nr.: 05-5502) was used. In short, yong leaves of this *L. chinense* individual used for the whole genome sequencing together with young leaves of *Vinca major* were first "chopped" with a sharp razor blade in 500µl Extraction Buffer (ice-cold), in a plastic petri disc. After 30-60 seconds of incubation, 2.0 ml Staining Buffer is added. This buffer contains Propidium Iodide (PI) as fluorescent dye and RNA-se. To the buffer is also added 0,1% DTT (Dithiothreitol) and 1% Polyvinylpyrolidone. The copped solution, containing cell constituents and large tissue remnants, is passed through a nylon filter of 50 µm mesh size. After incubation of at least 30 minutes at room temperature, the filtered solution with stained nuclei is send through the flow cytometer CyFlow (Sysmex Partec GmbH). At least 3000 nuclei of the sample and the internal standard (*Vinca major*) were measured. The fluorescence of the stained nuclei, passing through the focus of the light beam of a 50 mW, 532 nm green laser, is measured by a photomultiplier and converted into voltage pulses. These voltage pulses are electronically processed to yield integral and peak signals and have been processed by a computer. Finally, the DNA content of this *L. chinense* individual used in genome sequencing is 3.7 pg/2c, which means that the genome size of this individual plant is estimated to be ~1,809 Mb[3].

1.6 *De novo* genome assembly

The *Liriodendron* genome was *de novo* assembled using FALCON

(https://github.com/PacificBiosciences/FALCON) based on PacBio long reads (only reads longer than 10 kb were corrected and assembled, the daligner's option: -D24 -t30 -h480 -e.75 -w8 -l3000 -s1000 -k18). Errors in the PacBio reads were corrected within the FALCON pipeline. The assembled genome was firstly polished by Arrow whichi is from SMRT Link v5.0.0 based on raw PacBio data (--minConfidence 40 --minCoverage 5) and then paired-end Illumina reads of short-insert libraries (170bp, 250 bp, 500 bp and 800bp) were aligned to the assembly by BWA-mem v0.7.17 for a Pilon v1.21 correction[4] to improve assembly with these aligned results. Hybrid scaffolds with assembled contigs and optical genome maps were created by Bionano Access pipeline (https://bionanogenomics.com/support-page/bionano-access/) using merge P-value of 1×10^{-10} and alignment length of 60 bp. Based on the super-scaffolds, we utilized PBJelly v15.8.24[5] to do gap filling with the PacBio reads which corrected by Falcon before with the option '<blasr>-minMatch 8 -minPctIdentity 75 -bestn 1 -nCandidates 20 -maxScore -500 -nproc 4 -noSplitSubreads</blasr>' for protocol file. This Whole Genome Shotgun project has also been deposited under the same BioProject with an accession number PRJNA418360.

1.7 Linkage map construction

A total of 150 F1 seedlings, segregating from a single cross using the parents 'Lushan' and 'NK', was used to construct the linkage map. These two parent individuals are planted in the Xiashu Tree Farm, Jiangsu, China, and the female parent 'Lushan' originated from Lushan, Jiangxi, China and the male parent 'NK' originated from South

California, USA. These 150 F1 seedlings are planted in Hubei, China. Linkage analysis was implemented by using JoinMap 4.0[6]. In the first step, RAD-based SNP markers were selected according to the expected segregation ratio, such as two heterozygous SNP markers between two parents were expected to segregate at a 1:2:1 ratio, and one heterozygous and one homozygous SNP allele between two parents were expected to segregate at a 1:1 ratio. Subsequently, Distorted markers (Po0.01) were filtered to construct a genetic map by using a chi-square test. Finally, the candidate markers were divided into 19 linkage groups (Supplementary Fig. 2). Then, reads that contained SNP markers were aligned to the scaffolds. All these SNP markers were used to construct the linkage map with the CP population model in JoinMap (Supplementary Table 6).

1.8 Construction of BAC libraries

Nuclei were isolated from 200 grams of etiolated young leaves as described as by Peterson *et al.*[7] and Zhang *et al.*[8]. High molecular weight (HMW) DNA was released from nuclei by proteinase K in lysis buffer (0.1 mg/mL Proteinase K dissolved in 0.5M EDTA, PH = 9.1) at 50 °C for 48 hours. Lysis buffer was exchanged after 24 hours during a 48-hour period. Plugs (usually containing 5-6 μg undigested HMW DNA) were partially digested with BamHI or HindIII. After digestion, size selection was first carried out by PFGE separation for 16 h with a setting of 6 V/cm, pulse time 1-40 s, 12.5 °C, angle 120 °, then for 16 h with a setting of 6 V/cm, pulse time 3-5 s, 12.5 °C, angle 120 ° in 0.25× TBE buffer. We harvested agarose gels, containing DNA fragments with a size range of 200 to 400 kb, and performed DNA elution with 350-

450 μl 1× TAE buffer using a Bio-Rad model 422 Electro-Eluter (Bio-Rad, USA). Eluted DNA was ligated into pIndigoBAC-5 vectors (Epicentre, USA). The mol ratio of vector to insert DNA was 10:1. The ligation products were introduced into ElectroMAXTM DH10BTM cells (Invitrogen, USA) via the Gene Pulser XcellTM Total System (Bio-Rad, USA) at 1.7 kV/cm, 200 Ω with a 0.1 cm cuvette (Bio-Rad, USA). Transformed cells were spread on LB Petri plates containing 12.5 μg·mL-1 chloramphenicol, 0.55 M IPTG and 80 μg X-GAL/ml[9]. White clones were picked with sterile toothpicks manually and arranged in 384-well plates, which were then filled with 80 μl ice-cold LB media containing 12.5 μg·mL-1 chloramphenicol. All 384-well plates were incubated at 37 °C overnight until the media became muddy cloudy. Clones in 384-well plates were kept in -80 °C.

1.9 Genome assembly assessment

We used a 500-bp sliding window to calculate GC content and average sequencing depth using the *L. chinense* genome assembly as a reference. Usually, genomic regions with high or low GC content will possess a low sequencing depth compared to a median GC content region. Our results indicated there were no obvious sequence biases or contaminations. To access the integrity of the *L. chinense* assembly, we aligned about 70× (i.e. ~119 Gb) paired-end reads from the 170 bp genomic libraries onto the *L. chinense* assembly using SOAPdenovo v2.04 with the parameters set to "-m 127 -x 190 –v 5 -l 32 -s 40", resulting in a mapping rates of 88.78%.

We also assessed the genome assembly by using BAC sequencing. Those 89 BAC sequences were mapped back to the assembled reference genome by BLASTN with an E-value of 1e-5. Subsequently, solar was utilized to conjoin fragmental alignments for each BAC alignment result. We found that 99.75% of the BAC sequences were covered without any obvious misassemblies (Supplementary Fig. 4).

A total of 14 Mb PE RNA-Seq reads from Hiseq 2000 sequencing libraries, representing expressed sequences from 4 different *L. chinense* tissues (i.e., sepal, bud, stamen and stigma), was assembled with Trinity v2.4.0[10]. All assembled unigenes were further used for evaluating the completeness of the *L. chinense* genome assembly based on BLAT v35 with default parameters. These results showed that the assembly covered 99.78% of the 66,934 unigenes and 91.89% of these unigenes could be mapped to the assembly with >90% sequence in one scaffold (Supplementary Table 8).

The 1440 conserved plant genes from the BUSCOs[11] database were also mapped back to the genome assembly by BLAT to calculate the gene region; 1,300 (90.28%) conserved plant genes could be found in the assembled genome. (Supplementary Table 9).

1. Genome annotation

1.1 Repeat annotation

Genome annotation was performed based on the genome version PVNU01000000. We identified tandem repeats and transposable elements (TEs) separately. Tandem repeats were predicted using Tandem Repeats Finder 4.04[12] with the following parameters:

"Match = 2, Mismatch = 7, Delta = 7, PM = 80, PI = 10, Minscore = 50, and MaxPeriod = 2000".

TEs were identified in the genome using a combination of homology-based and *de novo* approaches. For the homology based approach, we first identified known TEs using RepeatMasker against the Repbase 16.10[13] database of known repeat sequences, and then used RepeatProteinMask, implemented in RepeatMasker, to identify TEs by aligning the genome sequence to the TE protein database. For the *de novo* approach, we constructed a repeat library generated by RepeatModeler v1.0.11[14] with default parameters, obtaining consensus sequences and classification information for each repeat family. Then RepeatMasker was run on the genome sequences, using the RepeatModeler consensus sequence as the library.

Finally, all repeat sequences identified by the different methods were combined into the final repeat annotation (Supplementary Tables 10-12).

1.2 Gene prediction

Gene model prediction was conducted by the MAKER pipeline (version 2.31.10)[15], integrating *ab initio* prediction with *de novo* assembled transcripts from short-read mRNA sequencing, isoform-sequencing full-length transcripts, and protein homology data. A high-confidence gene model was conducted by further removing transposons and low-confidence predictions.

1.3 Gene annotation

Gene functionality was predicted based on the best match derived from alignments to proteins annotated in SwissProt and TrEMBL databases[16] using blastp v2.3.0[17] (E-value $\leq$ 10-5). Motifs and domains were annotated using InterProScan[18] by searching against publicly available protein databases, including Pfam[19], PRINTS[20], PROSITE[21], ProDom[22], and SMART[23]. Descriptions of gene products, i.e., Gene Ontology (GO) terms, were retrieved from the corresponding InterPro entries. We also mapped the *Liriodendron* reference genes to KEGG[24] pathway maps by searching KEGG databases and finding the best hit for each gene. Finally, 29,482 genes (83.59% of all predicted genes) were functionally annotated and the remaining 5,787 genes, with no functional annotation, were labeled "hypothetical proteins" (Supplementary Table 7).

1.4 ncRNA annotation

A non-coding RNA (ncRNA) is any RNA molecule that is not translated into a protein. Here, four types of non-coding RNAs (ncRNAs), including micro RNAs (miRNAs), transfer RNAs (tRNAs), ribosomal RNAs (rRNAs), and small nuclear RNAs (snRNAs), were annotated. tRNA genes were predicted based on tRNAscan-SE v1.3.1[25] with parameters chosen for eukaryotes. If more than 80% of the length of a tRNA gene was covered by SINE TEs, then it was defined as SINE masked. rRNA fragments were identified by aligning human's rRNA sequences to the *Liriodendron* genome by using BLASTN[17] with a parameter of E value $\leq$1e-5, identity $\geq$85% and matched length $\geq$50bp. miRNA and snRNA genes were detected by using INFERNAL[26] software

(version 1.1.2) against the Rfam database[27] (Release 9.1), with Rfam's family-specific "gathering" cutoff.

2. Genome evolution

2.1 Whole genome duplication

To identify syntenic blocks, protein sequences from *L. chinense* and grape were first blasted against themselves using BLASTP[17]. Then these results were subjected to MCscan v0.8[28] to determine syntenic blocks, defining five genes as being required to define a synteny block. We then calculated the 4DTv (fourfold degenerate synonymous sites of the third codon) for syntenic segments from the concatenated alignments, constructed by fourfold degenerate sites of all gene pairs found in each segment, and plotted the distribution of the 4DTv values. One peak around 0.25 was observed in the *L. chinense* genome (Supplementary Fig. 8). An all-against-all comparison based on protein sequences was performed on *L. chinense* using BLASTP 2.2.29[17] with an E value of 10-5. Then alignments were further filtered to retain pairs for which the shorter sequence was at least 50% of the longer sequence, and the alignment was at least 50% of the shorter sequence. If one sequence had multiple matches meeting the cut-offs, these were grouped into a paralogue group, including any other genes that were associated with these matches. Next, all possible pairs of protein sequences within each group were aligned using MUSCLE 3.8.31[29] with default parameters. A nucleotide alignment was generated from the protein alignment using a Python script. Synonymous substitutions were estimated using the codeml program from PAML 4.8[30].

The Ks scores within each group were then corrected to remove redundant values; only those representing duplication events within the group were retained (in a group of n genes, there are n - 1 possible duplication events) using the method described in previous studies[31,32]. Moreover, another K_s method which was development by Maere *et al.*[33] was used to interpret the results. Based on the previously obtained blast results, some pairs were filter based on an E value cutoff of e-10, after which gene families were built with the OrthoMCL version 5[34]. Each gene family was aligned by PRANK[35], and K_s were estimated for all pairwise comparisons within a gene family by the CODEML program of the PAML package[30]. Gene families were then subdivided into subfamilies for which K_s estimates between members did not exceed a value of 5. To correct for the redundancy of Ks values (a gene family of n members produces $n(n–1)/2$ pairwise Ks estimates for n-1 retained duplication events), for each subfamily a phylogenetic tree was constructed using PhyML 3.0[36] under default settings. Subsequently, each tree was rooted by treebest. For each duplication node in the resulting phylogenetic tree, all m K_s estimates between the two child clades were added to the K_s distribution with a weight of $1/m$, so that the weights of all K_s estimates for a single duplication event sum up to one. The K_s-based relative age distributions were constructed for both of the genome (Fig 1a) and transcriptome (Fig 1b).

2.2 LTR insertion

Based on the repeat annotation, we counted the content and distribution of TEs in the *Liriodendron* genome using R program. Among the TEs, long terminal repeats (LTRs)

were the most abundant and occupied 56.25% of the genome, while DNA transposons occupied 5.81% and long interspersed nuclear elements (LINEs) occupied 1.70% (Supplementary Table 11). Within LTRs, the *Gypsy* superfamily was more abundant than the *Copia* superfamily (Supplementary Table 12 and Supplementary Fig. 9). In addition, TEs within the *Liriodendron* genome are located in four regions: the intergenic regions (84.71%), gene regions (13.93%), Proximal promoter (with less than 3,000 bp from its adjacent gene 5' end, 0.73%) and Proximal 3' end (with less than 3,000 bp from its adjacent gene 3' end, 0.64%) (Supplementary Fig. 10).

As the genome size of *L. chinense* is about 1.7G, we investigated the effect of genome expansion on LTR presence (Supplementary Fig. 13). All the LTRs sequences were identified with complete 5'LTR and 3'LTR by the LTR-STRUC program under the default p. Each of the 5' LTR flank sequences and 3' flank sequences were aligned by MUSCLE[29]. Then, the distance of the alignment sequences was calculated by the disMat. The insert time was calculated using the following formula: T=K/2r. Assuming an intergenic nucleotide substitution rate that was roughly twice as slow in genic regions, a substitution rate of 1.51×10^{-9} per site per year was used to convert LTR sequence divergence values into the estimated insertion time.

3. Genome phylogeny

3.1 orthologue identification

Ortholog groups (OGs) were constructed using 17 other land plants: six eudicots (*Arabidopsis thaliana*, *Populus trichocarpa*, *Vitis vinifera*, *Coffea canephora*, *Ipomoea*

nil and *Fraxinus excelsior*); six monocots (*Brachypodium distachyon*, *Xerophyta viscosa*, *Asparagus officinalis*, *Musa acuminata*, *Ananas comosus* and *Oryza sativa*); three magnoliids (*Magnolia Grandiflora*, *Michelia alba* and *Persea americana*); one basal angiosperm (*Amborella trichopoda*); and one gymnosperm (*Gnetum montanum*) by using the software OrthoFinder v2.2.3[37]. Most of these plant species have genome data except for three magnoliids plants in which transcriptome data were used in this study. Among these three magnoliids, *Magnolia Grandiflora*, *Michelia alba* and *Persea americana*, the first two were sequenced in this study and the last one was available in Ibarra-Laclette *et al.*[38]. To obtain as many genes as possible, we sequenced the mixed tissues comprised of flowers, stems and leaves in both two Magnoliaceae plants and the resting Lauraceae plant we selected was also sequenced based on mixed tissues comprised of seeds, roots, stems, leaves, aerial buds and flowers[38]. The final numbers of available protein sequences of these three magnoliids, *Magnolia Grandiflora*, *Michelia alba* and *Persea americana*, were 33,943, 34,672 and 46,351, respectively. First, we performed OG construction using OrthoFinder[37]. Then, we selected low-copy OGs with the number of putative orthologues less than two in each species and putative orthologues were found in at least four eudicots, four monocots, three magnnliids, one basal angiosperm and one gymnosperm.

After that, a total of 1,163 low-copy OGs were separately aligned using Clustal Omega v1.2.4[39] and all alignments were further trimmed by using TrimAl 1.2[40]. Next, we constructed 1,163 single-gene trees by using RAxML v8.2.11[41] with the PROTCATWAG mode. Finally, we rooted each OG tree using *Gnetum montanum* and

compared these single-gene trees with the species tree (Supplementary Fig. 14) after masking all magnoliids. Due to the limited number of informative sites in one gene, it was hard to use a single-gene tree to resolve the relationship among the low-level taxonomic hierarchies. Therefore, we selected the OGs with genes that, as they should, formed a monophyletic gene clade within species of a monophyletic organismal group (that is, eudicots and monocots) and the only one basal angiosperm, *Amborella*, was sister to the clade of monocots and eudicots. After that, we unmasked all magnoliids plants and excluded OGs with different magnoliids plants clustered with different clades, that is eudicots, monocots and the clade of monocots and eudicots. In other words, we only selected OGs with all magnoliids plants clustered with the same clade (see examples in Supplementary Fig. 15), ultimately resulting in 502 low-copy OGs. Finally, we classified these OGs according to which clade the magnoliids clustered with into a sister group, ultimately resulting in three alternative topologies.

3.2 Phylogenetic signal quantification

We calculated phylogenetic signal as described in Sheng *et al.*[42]. Simply, we first calculated the site-wise log-likelihood scores for the ML tree constrained to three alternative topologies. Then, we calculated the difference in site-wise log-likelihood scores (ΔSLS) between these three alternative topologies for every site. Next, by summing the ΔSLS scores of all sites, we could obtain the difference in gene-wise log-likelihood scores (ΔGLS) between three alternative topological hypotheses. After that, we could quantify the distribution of phylogenetic signal for these three alternative

phylogenetic topologies at the gene level, that is, we could count the number of genes supporting for each alternative topology. Among the 506 low-copy OGs, 166 supported the topology I, 167 supported the topology II and the final 169 OGs supported the topology III with no statistically significant difference (Supplementary Fig. 16).

In addition, we also excluded the OGs with ΔGLS values being outlier. The outlier ΔGLS values were well defined[31] and we calculated the upper whisker and the lower whisker and excluded the OGs with absolute ΔGLS values greater than the upper whisker or smaller than the lower whisker, resulting in 481 low-copy OGs with 157 OGs supporting topology I, 159 OGs supporting topology II and the final 165 OGs supporting topology III (Fig. 2b), showing an equal distribution of phylogenetic signal for each topology at gene level.

3.3 Species tree estimation

We estimated the phylogenetic tree based on these 502-OG gene trees and 481-OG gene trees using ASTRAL 5.6.1[43] (Supplementary Fig. 17). In addition, we also extracted and concatenated 78 genes from chloroplast genomes of 24 species for phylogenetic analysis (Supplementary Fig. 18).

3.4 Divergence time estimation

CDS sequences of 235 single-copy OGs constructed using 11 land plant: *A. thaliana*, *P. trichocarpa*, *Eucalyptus grandis*, *V. vinifera, B. distachyon*, *Elaeis guineensis*, *Phalaenopsis equestris* and *Spirodela polyrhiza, A. trichopoda* and the outgroup *Picea*

abies, were used for divergence time estimation based on the phylogenetic tree. The PAML MCMCTREE[30] performs Bayesian estimation of species divergence times using soft fossil constraints[44] under various molecular clock models. We incorporated three fossil constraints, i.e., *A. thaliana* - *P. trichocarpa* divergence (97-109 Mya), *E. grandis* - *V. vinifera* divergence (105-115 Mya) and Eudicots - monocots divergence (130-200 Mya)[45]. The program needs input files including a sequence alignment (nucleotide or protein), a phylogenetic tree with fossil calibrations, and a control file (usually called mcmctree.ctl) that contains the instructions for the program. The Markov chain Monte Carlo (MCMC) process of the PAML mcmctree was set to sample 1,000,000 times, with the sample frequency set to 50, after a burn-in of 5,000,000 iterations. Parameters of "finetune" were set at "0.004, 0.016, 0.01, 0.10, 0.58". Other parameters were set at default values.

3.5 Eudicot- and monocot-specific gene families

We achieved 114 eudicot- and 93 monocot-specific gene families from Monocot PLAZA 3.0[46] (Supplementary Fig. 19) and identified homologous genes present in *Amborella* and *Liriodendron* using BLASTP[17] with parameters set to: E value ≤1e-5, identify ≥40% and coverage ≥60%. We then counted the number of eudicot- and monocot-specific gene families contained in the *Amborella* (29 and 16 respectively) and *Liriodendron* (52 and 31 respectively) genomes. Furthermore, we performed a chi-square test to check the difference between the ratio of eudicot- versus monocot-specific gene families in *Liriodendron* (52/31) and that in *Amborella* (29/16), resulting

in a χ^2 of 0.1166 (p-value = 0.7328), showing no significant difference. We also performed this analysis on a monocot plant *Spirodella polyrhiza* (a ratio of 15/25) and a eudicot plant *Macleaya cordata* (a ratio of 78/19) which resulted in a χ^2 of 15.691 (p-value = 0.0003708) and χ^2 of 10.7940 (p-value = 0.0010), both showing a significant bias (Fig. 2c).

3.7 Gene family expansion and contraction

We used Café v4.0.1[47], a random birth and death model proposed to study gene gain and loss in gene families across a user-specified phylogenetic tree, to identify gene families that had undergone expansion or contraction across the ML tree that was constructed based on the 235-gene data set. Usually, a global parameter λ (lambda), which describes both gene birth (λ) and death (μ, equal to -λ) rate across all branches in the tree for all gene families is estimated using maximum likelihood. Then, a conditional *p*-value is calculated for each gene family, and families with a conditional *p*-value less than the threshold (0.05) will be considered as having an accelerated rate of gain and loss. Also, branches responsible for a low overall *p*-value of significant families will be identified.

4. Resequencing

4.1 Plant materials used for resequencing

To evaluate a broader range of genetic diversity between the two *Liriodendron* species and to compare their respective population structures, resequencing was conducted in

20 accessions covering a wide range of genetically and phylogenetically diverse materials. DNA from 14 *L. chinense* and six *L. tulipifera* adult plants was extracted using a modified CTAB protocol[1]. Paired-end libraries with insert sizes of 100-150 bp were constructed according to the manufacturer's instruction (Illumina, San Diego, CA, USA) and sequenced by Illumina sequencing technology at Illumina technology at Beijing Genome Institute, Shenzhen (BGI-Shenzhen, China). Whole genome resequencing of 20 *Liriodendron* plants generated from 15.14 Gbp to 72.6 Gbp nucleotides of sequence with an average depth of 39.4× (Supplementary Table 15). Sequences have been deposited in the NCBI Sequence Read Archive under project PRJNA418361. In addition, natural distribution maps of *L. chinense*[48] and *L. tulipifera* were plotted in R using the package ggmap[49] (Supplementary Fig. 20).

4.2 SNP calling

Paired-end reads (100bp or 150bp) obtained from sequencing were mapped to the *de novo* genome with BWA[50]. After the alignment, results in the SAM file format were converted to bam format using SAMtools v1.3.1[51]. These bam files were sorted and duplicated reads were marked by Picard pack tools. SNP detection was carried out by the Genome Analysis Toolkit (GATK, version 3.2.2) [52]. As there is a low-quality alignment around an indel region, two steps of realignment were implemented in GATK: the RealignerTargetCreator package was used to identify regions which need realignment in the first step. Then the IndelRealigner performed realignment of regions found in the first step. SNP calling was performed with UnifiedGenotyper and Samtools

mpileup, then SelectVariants was used to combine the raw vcf files as dbSNP, which was created by SAMtools and UnifiedGenotyper, filtering raw SNPs with “QD <20.0 or ReadPosRankSum <-8.0 or FS >10.0 or QUAL <meanqual”. After that, base-quality score recalibration was performed with BaseRecalibrator and the realigned bam file was reduced by PrintReads and ReduceReads. In the next step CombineVariants was used to combine the individual Gvcf files into a combind population of vcf files as a dbSNP. Based on the dbSNP data and the BaseRecalibrator BAM files, GATK was used to call raw SNPs and indels using parameters from UnifiedGenotyper. After obtaining the raw result, VQSR, then VariantFiltration were used to filter some low-quality SNPs with “QD <2.0, MQ <40. 0, ReadPosRankSum <-8.0, FS >60.0, HaplotypeScore >13.0 and MQRankSum <-12.5”. Missing SNP sites were filtered and then used for analysis in the next step. SNPs were annotated by SNPEFF[53] and summarized by a customized Perl script. The annotation for the complete SNPs set was used for subsequent positive selection analysis.

4.3 Phylogenetic and population structure analysis

SNPs were used to construct a phylogenetic tree, based on the neighbor-join method by TreeBeST v1.9.2[54] (Fig. 3b) and the Maximum likelihood method by RAxML[55] (Supplementary Fig. 23). The resulting phylogenetic trees inferred by these two methods are about the same, excepting the position of the DBS provenance. In the NJ tree, all *L. chinense* individuals from China West clustered together (the CW group) and the rest of the *L. chinense* collected from China East clustered into the second group (the CE

group). LY came from a provenance geographically located in the transition region between western and eastern China and did not cluster into any group. The third group (the NA group) was comprised of all *L. tulipifera* individuals collected from North America. In the ML tree, DBS did not cluster into the east group of China and was positioned the same as LY. Intriguingly, DBS is geographically close to LY. In general, both NJ and ML trees clustered these *Liriodendron* individuals into three main geographical groups. In addition, ped files were created as input for PLINK version 1.07 with parameters "--ped ped_file --recode12 --geno 0.5 --map output_map". Then the program FRAPPE v1.1[56] was utilized to infer population structure and ancestry information. The analysis was based on 13.3M SNP sites and we did not assume any prior information about their ancestry. We ran 10,000 iterations and pre-defined the number of clusters, K, from 2 to 5. ADMIXTURE v1.3.0[57] was used to find the best K value based on a cross-validation test. We performed a PCA following the procedure as reported. The eigenvector decomposition of the transformed genotype data was performed using the R function eigen, and the significance of the eigenvectors was determined with a Tracey-Widom test, implemented in the program twstats, provided by EIGENSOFT 3.2[58].

Nucleotide diversity (π)[59] and the Watterson estimator (θ_w)[60] were used to measure the degree of variability within a population or species[61]. F_{st} was used to measure the population differentiation and genetic distance, based on genetic polymorphism data. π, θ_w and F_{st} were calculated on the basis of the genotype of each line at each SNP position using BioPerl.

4.4 PSMC analysis

The PSMC model, originally applied to human genomes[62], after which it was also applied to plant genomes[63,64], was used to study the effective population size (N_e) of the two *Liriodendron* species over time. PSMC inferred the local time since the most recent common ancestor on the basis of the local density of heterozygotes by use of a hidden Markov model in which the observation is a single diploid sequence[62]. PSMC utilizes sequence reads that are mapped to a reference genome to estimate historical fluctuations in N_e. To scale PSMC results to real time, we assumed 6 years per *Liriodendron* generation (g) and a per-generation mutation rate (μ) of 7×10^{-9}. PSMC was otherwise conducted using default parameters.

For all *L. chinense*, the first bottleneck occurred about 0.9 million years ago (Fig. 4), during the Xixiabangma Glaciation, around 1.17-0.8 million years ago[65]. The high mass accumulation rate (MAR) of Chinense loess[66] during that time indicates a cold and dry climatic period. Then, the *L. chinense* population started to expand until to its peak about 0.3-0.4 million years ago, just during an interglacial stage with warm weather as evidenced by low MAR. Then, along with the beginning of the Guxiang Glaciation (i.e., Penultimate Glaciation, 0.3-0.13 million years ago)[65], the *L. chinense* population declined rapidly and arrived at its next bottleneck around the time the Baiyu (the Last) Glaciation occured (0.07-0.01 million years ago)[65]. The *L chinense* population always remained at a very low estimated N_e in this bottleneck, either during the warm Greatest Lake Period (30,000-40,000 years ago) or after retreat of the Quaternary glaciation

(after 20,000 years ago), indicating that *L chinense* might have migrated and been restricted to its glacial refugia, widely scattered in eastern Asia.

For *L. tulipifera*, there was a sustained decrease of population since the Late Miocene (Fig. 4). The population bottleneck occurred approximately 0.2 million years ago, around the time of Penultimate Glaciation. Then, the population of *L. tulipifera* experienced a period of explosive growth and achieved its peak during the warm Greatest Lake Period (30,000-40,000 years ago), after which it stayed stable.

References

1. Murray, M.G. & Thompson, W.F. Rapid isolation of high molecular weight plant DNA. *Nucleic Acids Res* **8**, 4321-5 (1980).
2. Mayjonade, B. *et al.* Extraction of high-molecular-weight genomic DNA for long-read sequencing of single molecules. *Biotechniques* **61**, 203-205 (2016).
3. Dolezel, J., Bartos, J., Voglmayr, H. & Greilhuber, J. Nuclear DNA content and genome size of trout and human. *Cytometry A* **51**, 127-128 (2003).
4. Walker, B.J. *et al.* Pilon: an integrated tool for comprehensive microbial variant detection and genome assembly improvement. *PLoS One* **9**, e112963 (2014).
5. English, A.C. *et al.* Mind the gap: upgrading genomes with Pacific Biosciences RS long-read sequencing technology. *PLoS One* **7**, e47768 (2012).
6. Van Ooijen, J.W. *JoinMap 4: Software for the Calculation of Genetic Linkage Maps in Experimental Populations*, (Kyazma, 2006).
7. Peterson, D.G., Tomkins, J.P., Frisch, D.A., Wing, R.A. & Paterson, A.H. Construction of plant bacterial artificial chromosome (BAC) libraries: an illustrated guide. *Journal of Agricultural genomics* **5**, 1-100 (2000).
8. Zhang, H.B., Zhao, X., Ding, X., Paterson, A.H. & Wing, R.A. Preparation of megabase-size DNA from plant nuclei. *The Plant Journal* **7**, 175-184 (1995).
9. Sambrook, J., Fritsch, E.F. & Maniatis, T. *Molecular cloning: a laboratory manual*, (Cold spring harbor laboratory press, 1989).
10. Grabherr, M.G. *et al.* Full-length transcriptome assembly from RNA-Seq data without a reference genome. *Nat Biotechnol* **29**, 644-52 (2011).
11. Simao, F.A., Waterhouse, R.M., Ioannidis, P., Kriventseva, E.V. & Zdobnov, E.M. BUSCO: assessing genome assembly and annotation completeness with single-copy orthologs. *Bioinformatics* **31**, 3210-3212 (2015).
12. Benson, G. Tandem repeats finder: a program to analyze DNA sequences. *Nucleic Acids Res* **27**, 573-80 (1999).
13. Jurka, J. *et al.* Repbase Update, a database of eukaryotic repetitive elements. *Cytogenet Genome Res* **110**, 462-7 (2005).

14. Price, A.L., Jones, N.C. & Pevzner, P.A. De novo identification of repeat families in large genomes. *Bioinformatics* **21 Suppl 1**, i351-8 (2005).
15. Cantarel, B.L. *et al.* MAKER: an easy-to-use annotation pipeline designed for emerging model organism genomes. *Genome research* **18**, 188-196 (2008).
16. Bairoch, A. & Apweiler, R. The SWISS-PROT protein sequence database and its supplement TrEMBL in 2000. *Nucleic Acids Res* **28**, 45-48 (2000).
17. Altschul, S.F., Gish, W., Miller, W., Myers, E.W. & Lipman, D.J. Basic local alignment search tool. *J Mol Biol* **215**, 403-10 (1990).
18. Zdobnov, E.M. & Apweiler, R. InterProScan--an integration platform for the signature-recognition methods in InterPro. *Bioinformatics* **17**, 847-848 (2001).
19. Finn, R.D. *et al.* The Pfam protein families database: towards a more sustainable future. *Nucleic Acids Res* **44**, D279-285 (2016).
20. Attwood, T.K. *et al.* The PRINTS database: a fine-grained protein sequence annotation and analysis resource--its status in 2012. *Database (Oxford)* **2012**, bas019 (2012).
21. Sigrist, C.J. *et al.* PROSITE, a protein domain database for functional characterization and annotation. *Nucleic Acids Res* **38**, D161-166 (2010).
22. Bru, C. *et al.* The ProDom database of protein domain families: more emphasis on 3D. *Nucleic Acids Res* **33**, D212-215 (2005).
23. Letunic, I., Doerks, T. & Bork, P. SMART: recent updates, new developments and status in 2015. *Nucleic Acids Res* **43**, D257-260 (2015).
24. Kanehisa, M. & Goto, S. KEGG: kyoto encyclopedia of genes and genomes. *Nucleic Acids Res* **28**, 27-30 (2000).
25. Lowe, T.M. & Eddy, S.R. tRNAscan-SE: a program for improved detection of transfer RNA genes in genomic sequence. *Nucleic Acids Res* **25**, 955-64 (1997).
26. Nawrocki, E.P., Kolbe, D.L. & Eddy, S.R. Infernal 1.0: inference of RNA alignments. *Bioinformatics* **25**, 1335-7 (2009).
27. Griffiths-Jones, S. *et al.* Rfam: annotating non-coding RNAs in complete genomes. *Nucleic Acids Res* **33**, D121-4 (2005).
28. Wang, Y. *et al.* MCScanX: a toolkit for detection and evolutionary analysis

of gene synteny and collinearity. *Nucleic Acids Res* **40**, e49 (2012).
29. Edgar, R.C. MUSCLE: multiple sequence alignment with high accuracy and high throughput. *Nucleic Acids Res* **32**, 1792-7 (2004).
30. Yang, Z. PAML 4: phylogenetic analysis by maximum likelihood. *Mol Biol Evol* **24**, 1586-91 (2007).
31. Maere, S. *et al.* Modeling gene and genome duplications in eukaryotes. *Proc Natl Acad Sci U S A* **102**, 5454-9 (2005).
32. Blanc, G. & Wolfe, K.H. Widespread paleopolyploidy in model plant species inferred from age distributions of duplicate genes. *Plant Cell* **16**, 1667-78 (2004).
33. Maere, S. *et al.* Modeling gene and genome duplications in eukaryotes. *Proc Natl Acad Sci U S A* **102**, 5454-5459 (2005).
34. Li, L., Stoeckert, C.J., Jr. & Roos, D.S. OrthoMCL: identification of ortholog groups for eukaryotic genomes. *Genome Res* **13**, 2178-89 (2003).
35. Loytynoja, A. Phylogeny-aware alignment with PRANK. *Methods Mol Biol* **1079**, 155-70 (2014).
36. Guindon, S. *et al.* New algorithms and methods to estimate maximum-likelihood phylogenies: assessing the performance of PhyML 3.0. *Syst Biol* **59**, 307-21 (2010).
37. Emms, D.M. & Kelly, S. OrthoFinder: solving fundamental biases in whole genome comparisons dramatically improves orthogroup inference accuracy. *Genome Biol* **16**, 157 (2015).
38. Ibarra-Laclette, E. *et al.* Deep sequencing of the Mexican avocado transcriptome, an ancient angiosperm with a high content of fatty acids. *BMC Genomics* **16**, 599 (2015).
39. Sievers, F. & Higgins, D.G. Clustal Omega for making accurate alignments of many protein sequences. *Protein Sci* **27**, 135-145 (2018).
40. Capella-Gutierrez, S., Silla-Martinez, J.M. & Gabaldon, T. TrimAl: a tool for automated alignment trimming in large-scale phylogenetic analyses. *Bioinformatics* **25**, 1972-3 (2009).
41. Stamatakis, A. RAxML-VI-HPC: maximum likelihood-based phylogenetic analyses with thousands of taxa and mixed models. *Bioinformatics* **22**, 2688-90 (2006).

42. Shen, X.X., Hittinger, C.T. & Rokas, A. Contentious relationships in phylogenomic studies can be driven by a handful of genes. *Nat Ecol Evol* **1**, 126 (2017).
43. Zhang, C., Sayyari, E. & Mirarab, S. ASTRAL-III: Increased Scalability and Impacts of Contracting Low Support Branches. 53-75 (2017).
44. Yang, Z. & Rannala, B. Bayesian estimation of species divergence times under a molecular clock using multiple fossil calibrations with soft bounds. *Molecular Biology and Evolution* **23**, 212-226 (2006).
45. Kumar, S., Stecher, G., Suleski, M. & Hedges, S.B. TimeTree: A Resource for Timelines, Timetrees, and Divergence Times. *Mol Biol Evol* **34**, 1812-1819 (2017).
46. Proost, S. *et al.* PLAZA 3.0: an access point for plant comparative genomics. *Nucleic Acids Res* **43**, D974-81 (2015).
47. De Bie, T., Cristianini, N., Demuth, J.P. & Hahn, M.W. CAFE: a computational tool for the study of gene family evolution. *Bioinformatics* **22**, 1269-71 (2006).
48. Hao, R., He, S., Tang, S. & S., W. Geographical distribution of Liriodendron chinense in China and its significance. *Journal of Plant Resources and Environment (China)* **4**, 1-6 (1995).
49. Kahle, D. & Wickham, H. ggmap: Spatial Visualization with ggplot2. *R Journal* **5**, 144-161 (2016).
50. Li, H. & Durbin, R. Fast and accurate short read alignment with Burrows-Wheeler transform. *Bioinformatics* **25**, 1754-60 (2009).
51. Li, H. *et al.* The Sequence Alignment/Map format and SAMtools. *Bioinformatics* **25**, 2078-9 (2009).
52. DePristo, M.A. *et al.* A framework for variation discovery and genotyping using next-generation DNA sequencing data. *Nat Genet* **43**, 491-8 (2011).
53. Cingolani, P. *et al.* A program for annotating and predicting the effects of single nucleotide polymorphisms, SnpEff: SNPs in the genome of Drosophila melanogaster strain w1118; iso-2; iso-3. *Fly (Austin)* **6**, 80-92 (2012).
54. Vilella, A.J. *et al.* EnsemblCompara GeneTrees: Complete, duplication-aware phylogenetic trees in vertebrates. *Genome Res* **19**, 327-35 (2009).

55. Stamatakis, A. RAxML-VI-HPC: maximum likelihood-based phylogenetic analyses with thousands of taxa and mixed models. *Bioinformatics* **22**, 2688-2690 (2006).

56. Tang, H., Peng, J., Wang, P. & Risch, N.J. Estimation of individual admixture: analytical and study design considerations. *Genet Epidemiol* **28**, 289-301 (2005).

57. Alexander, D.H., Novembre, J. & Lange, K. Fast model-based estimation of ancestry in unrelated individuals. *Genome Res* **19**, 1655-64 (2009).

58. Price, A.L. *et al.* Principal components analysis corrects for stratification in genome-wide association studies. *Nat Genet* **38**, 904-9 (2006).

59. Nei, M. & Li, W.H. Mathematical model for studying genetic variation in terms of restriction endonucleases. *Proc Natl Acad Sci U S A* **76**, 5269-73 (1979).

60. Watterson, G.A. On the number of segregating sites in genetical models without recombination. *Theor Popul Biol* **7**, 256-76 (1975).

61. Tajima, F. Evolutionary relationship of DNA sequences in finite populations. *Genetics* **105**, 437-60 (1983).

62. Li, H. & Durbin, R. Inference of human population history from individual whole-genome sequences. *Nature* **475**, 493-496 (2011).

63. Ibarra-Laclette, E. *et al.* Architecture and evolution of a minute plant genome. *Nature* **498**, 94-8 (2013).

64. Amborella Genome, P. The Amborella genome and the evolution of flowering plants. *Science* **342**, 1241089 (2013).

65. Zheng, B.X., Xu, Q.Q. & Shen, Y.P. The relationship between climate change and Quaternary glacial cycles on the Qinghai-Tibetan Plateau: review and speculation. *Quaternary International* **97**, 93-101 (2002).

66. Sun, Y.B. & An, Z.S. Late Pliocene-Pleistocene changes in mass accumulation rates of eolian deposits on the central Chinese Loess Plateau. *Journal of Geophysical Research Atmospheres* **110**, D23101 (2005).

Supplementary Tables

Supplementary Table 1. Summary of library construction and sequencing of Illumina data.

Paired-end Libraries	Insert Size	Average Read Length (bp)	Total Clean /Raw Data (Gb)	Sequencing Depth (×)[a]	Physical Depth (×)
**Solexa Reads**	170 bp	100	118.98/130.82	67.83/74.58	57.66/63.39
	250 bp	150	45.83/53.39	26.13/30.44	21.78/25.37
	500 bp	100	83.98/94.38	47.88/53.81	119.70/134.53
	800 bp	100	78.32/88.82	44.66/50.64	178.64/202.56
	**Total**		327.11/367.41	186.5/209.47	377.78/425.85

[a]: We estimate the sequencing coverage by assuming the genome size to be 1.75 Gb.

Supplementary Table 2. Statistics of corrected PB reads.

Reads	Size (bp)	Number	Depth
subreads >= 2k	147,893,889,877	12,381,613	87
subreads >= 5k	139,096,142,067	9,817,481	81.82
subreads >= 10k	114,919,709,311	6,546,414	67.60
subreads >= 12k	101,694,888,173	5,344,942	59.82
subreads >= 15k	80,162,479,742	3,742,966	47.15
subreads >= 16k	72,869,462,837	3,272,275	42.86
subreads >= 20k	46,478,797,744	1,792,900	27.34
subreads >= 25k	24,351,603,157	795,790	14.32
subreads >= 30k	11,919,374,791	338,169	7.01
subreads >= 35k	5,412,158,385	135,834	3.18
subreads >= 40k	2,228,225,395	50,081	1.31

Supplementary Table 3. Statistics of Bionano optical maps.

	Number	Length (bp)
Total data		315,411,275,361
Total label	20,474,808	
Total molecule	1,546,266	
Molecule (label number > 6)	1,189,663	
Average label per molecule	13.24	
Density of label per 100kb	6.49	
Molecule length > 100kb	1,546,266	315,411,275,361
Molecule length > 150kb	893,335	235,559,291,832

Supplementary Table 4. Estimation of the *L. chinense* genome size based on 17 K-mer statistics.

k-mer	k-mer no.	Peak depth	Genome size	Used bases	Used reads	Depth
17	4,210,050,595	24	1,754,187,748	52,625,632,420	657,820,404	30

Supplementary Table 5. Summary of the *L. chinense* genome assembly.

	Contig		Scaffold	
	**Size (bp)**	**Number**	**Size (bp)**	**Number**
**N90**[a]	190,349	1,500	276,287	638
**N80**	483,159	953	1,192,516	375
**N70**	786,779	674	1,988,182	265
**N60**	1,090,133	487	2,855,213	192
**N50**	1,434,331	347	3,525,943	138
**Longest**	9,960,025		19,271,491	
**Total Size**	1,742,411,609		1,742,423,874	
**Total Number (>=1kb)**		4,624		3,711
**Total Number (>=10kb)**		4,242		3,329

[a]: Nxx length is the maximum length L such that xx% of all nucleotides lie in contigs (or scaffolds) of size at least L.

Supplementary Table 6. Summary of linkage map construction.

Linkage group	Anchoring markers (no.)	cM	Scaffolds (no.)	Size (bp)
**1**	142	178.5	33	96,007,009
**2**	133	190.6	33	99,473,975
**3**	111	198	36	96,689,308
**4**	97	149.17	38	76,263,199
**5**	93	126.4	21	63,449,182
**6**	96	154.04	29	65,192,789
**7**	104	200.3	26	87,369,336
**8**	75	119.15	25	64,408,360
**9**	79	134.36	17	58,375,695
**10**	85	118.1	24	68,352,314
**11**	71	108.4	21	70,397,449
**12**	66	93.16	30	69,840,316
**13**	67	112.55	32	75,054,824
**14**	67	118.5	38	74,942,525
**15**	72	127.4	41	56,984,379
**16**	49	44.6	27	67,893,758
**17**	54	97.7	19	54,703,317
**18**	63	115.3	17	63,323,318
**19**	52	95.3	22	57,223,572

Supplementary Table 7. Gene annotation in the *L. chinense* genome.

	Number	Percent (%)
**Total**	35,269	100.00
**Annotated**	29,482	83.59
**SwissProt**	22,530	63.88
**TrEMBL**	29,089	82.48
**InterPro**	28,080	79.62
**KEGG**	22,123	62.73
**Unannotated**	5,787	16.41

Supplementary Table 8. Assessment of the *L. chinense* genome assembly using RNA-seq data.

Dataset	Number	Total Length (bp)	Bases Covered by Assembly (%)	Sequences Covered by Assembly (%)	With >90% Sequence in one Scaffold		With >50% Sequence in one Scaffold	
					Number	Percent (%)	Number	Percent (%)
All	66,934	51,960,045	97.80	99.78	61,508	91.89	66,578	99.47
>200bp	66,934	51,960,045	97.80	99.78	61,508	91.89	66,578	99.47
>500bp	28,940	40,497,573	97.64	99.85	26,074	90.10	28,772	99.42
>1000bp	16,537	31,698,287	97.50	99.90	14,684	88.79	16,443	99.43

Supplementary Table 9. Assessment of the *L. chinense* genome assembly and annotation completeness using BUSCO.

Types of BUSCOs	Count	Ratio
**Complete BUSCOs**	1,300	90.28%
**Complete and single-copy BUSCOs**	1,190	82.64%
**Complete and duplicated BUSCOs**	110	7.64%
**Fragmented BUSCOs**	47	3.26%
**Missing BUSCOs**	93	6.46%

Supplementary Table 10. Prediction of repetitive sequences in the *L. chinense* genome.

Type	Repeat Size (bp)	% of Genome
**RepeatProteinMask**[a]	258,445,113	14.83
**RepeatMasker**[b]	236,234,135	13.56
**TRF**[c]	79,438,868	4.56
De novo[d]	1,039,699,474	59.67
**Total**[e]	1,111,834,359	63.81

[a] and [b]: RepeatProteinMask and RepeatMasker were used to identify repeats in the genome according to homology to identified repeat elements in Repbase.

[c]: TRF was used to predict tandem repeats.

[d]: RepeatMasker was used to identify *de novo* repeat elements in the genome according to results from Piler-DF, RepeatScout and LTR-FINDER.

[e]: Total repeat regions were identified after combining all repeats identified and removing redundancy.

Supplementary Table 11. Categories of TEs predicted in the *L. chinense* genome.

	RepBase TEs		TE Proteins		*De novo*		Combined TEs[a]	
	Length (Mbp)	% in Genome	Length (Mbp)	% in Genome	Length (Mbp)	% in Genome	Length (Mbp)	% in Genome
**DNA**	16.73	0.96	3.16	0.18	88.78	5.10	101.22	5.81
**LINE**	12.89	0.74	2.45	0.14	18.12	1.04	29.59	1.70
**SINE**	0.06	0	0	0	0.32	0.02	0.38	0.02
**LTR**	208.76	11.98	252.84	14.51	940.91	54.00	980.11	56.25
**Other**	0.002	0	0	0	0	0	0.002	0
**Unknown**	0	0	0	0	6.64	0.38	6.64	0.38
**Total[a]**	236.23	13.56	258.45	14.83	1,025.30	58.84	1,074.11	61.64

[a]: the total number of TEs was identified by combining all repeats identified through different methods. As there are some overlaps between different methods, the combined number of TEs is less than the sum of repeats identified by all methods.

Supplementary Table 12. Subcategories of TEs predicted in the *L. chinense* genome.

Classification		*L. chinense*	
Order	Superfamily	Length (Mb)	% of genome
**Class I Retrotransposon**			
LTR	*Gypsy*	704.67	40.45
	Copia	227.86	13.08
	ERV	3.05	0.18
	Caulimovirus	2.08	0.12
	other	42.45	2.44
LINE	*RTE*	8.14	0.47
	L1	19.57	1.12
	L2	0.41	0.02
	other	1.47	0.08
SINE	*tRNA*	0.16	0.01
	5S	1.12E-04	6.43E-06
	other	0.22	0.01
Unclassified		1.58E-03	8.84E-06
**Class II DNA transposon**			
TIR	*PIF*	2.95	0.17
	hAT	22.18	1.27
	TcMar	0.71	0.04
	EnSpm	51.00	2.93
	MuDR	2.14	0.12
	other	19.09	1.09
Crypton	*Crypton*	0.32	0.02
Helitron	*Helitron*	2.05	0.12
Maverick	*Maverick*	0.78	0.04
Unclassified		4.20E-04	1.56E-04
**Unknown**		6.64	0.38
**Total TEs**		1074.11	61.64

Supplementary Table 13. Statistical analysis of the distribution of three TE superfamilies in four *Liriodendron* genome regions.

	Copia		*Gypsy*		*LINE/L1*	
	Observed values	Predicted values	Observed values	Predicted values	Observed values	Predicted values
**Gene**	146,796	148,284.30	171,039	264,487.90	25,453	11,542.90
**Proximal Promoter**	3,714	38,502.40	7,026	68,674.95	204	2,997.14
**Proximal 3' End**	2,884	38,502.40	5,787	68,674.95	130	2,997.14
**Intergenic**	562,264	490,440.40	1,092,634	874,775.90	29,922	38,177.38

All these three TE superfamilies, i.e., *Copia*, *Gypsy*, *LINE/L1*, showed an uneven distribution throughout the *Liriodendron* genome with χ^2 values of 74,924, 200,220 and 23,896, respectively, and all p-values of zero. The blue colour indicates that the predicted value is bigger than the observed value, and the red colour indicates that the predicted value is smaller than the observed value.

Supplementary Table 14. A summary of research on the phylogentic relationships among magnoliids, eudicots and monocots.

Author	Year	Journal	Article	Gene			Non-coding sequence	Morphological characters	Species number	Gene number	Method	Software	Simplified topology	Classification	DOI
				nucleus	plastid	mitochondrion									
Mathews & Donoghue	1999	Science	The Root of Angiosperm Phylogeny Inferred from Duplicate Phytochrome Genes	PHYA, PHYC	-	-	-	-	26	2	concatenated, MP	PAUP* 4.0	((magnoliids, (monocots, eudicots):<50):86, basal angiosperm)	III	10.1126/science.286.5441.947
Soltis et al.	1999	Nature	Angiosperm phylogeny inferred from multiple genes as a tool for comparative biology	18S rDNA	atpB, rbcL	-	-	-	567	3	concatenated, MP	PAUP* 4.0	((eudicots, (monocots, magnoliids):56):71, basal angiosperm)	II	10.1038/46528
Qiu et al.	1999	Nature	The earliest angiosperms: evidence frommitochondrial, plastid and nuclear genomes	18S rDNA	atpB, rbcL	atp1, matR	-	-	105	5	concatenated, MP	PAUP*4.0b2	((monocots, (eudicots, magnoliids):<50):<50, basal angiosperm)	I	10.1038/46536
Barkman et al.	2000	PNAS	Independent and combined analyses of sequences from all three genomic compartments converge on the root of flowering plant phylogeny	18S rDNA	atpB, rbcL	atpA, matR, coxl	-	-	35	6	concatenated, NJ	PAUP*4.0b3	((monocots, (eudicots, magnoliids):<50):99, basal angiosperm)	I	10.1073/pnas.220427497
Graham & Olmstead	2000	American Journal of Botany	Utility of 17 chloroplast genes for inferring the phylogeny of the basal angiosperms	-	√	-	-	-	19	17	concatenated, MP	PAUP*	((magnoliids, (monocots, eudicots):25):76, basal angiosperm)	III	10.2307/2656749
Soltis et al.	2000	Botanical Journal of the Linnean Soiety	Angiosperm phylogeny inferred &om 18s rDNA, rbcL, and atpB sequences	18S rDNA	atpB, rbcL	-	-	-	567	3	concatenated, MP	RATCHET	((eudicots, (monocots, magnoliids)), basal angiosperm)	II	10.1m/b0j1.2000.0380
Qiu et al.	2000	International Journal of Plant Sciences	Phylogeny of Basal Angiosperms: Analyses of Five Genes from Three Genomes	18S rDNA	atpB, rbcL	atp1, matR	-	-	105	5	concatenated, MP	PAUP*4.0b2	((monocots, (eudicots, magnoliids):<50):<50, basal angiosperm)	I	10.1086/317584
Doyle & Endress	2000	International Journal of Plant Sciences	Morphological Phylogenetic Analysis of Basal Angiosperms: Comparison and Combination with Molecular Data	18S rDNA	atpB, rbcL	-	-	108	52	-	concatenated, MP	PAUP 3.1.1	((eudicots, (monocots, magnoliids):<50):63, basal angiosperm)	II	10.1086/317578
Sun et al.	2002	Science	Archaefructaceae, a New Basal Angiosperm Family	18S rDNA	atpB, rbcL	-	-	17	174	-	MP	-	((eudicots, (monocots, magnoliids)), basal angiosperm)	II	10.1126/science.1069439
Borsch et al.	2003	Journal of Evolutionary Biology	Noncoding plastid trnT-trnF sequences reveal a well resolved phylogeny of basal angiosperms	-	-	-	trnT-trnF	-	38	-	MP	PAUP*4.0b6	((monocots, (eudicots, magnoliids):<50):100, basal angiosperm)	I	10.1046/j.1420-9101.2003.00577.x
Qiu et al.	2005	International Journal of Plant Sciences	Phylogenetic analyses of basal angiosperms based on nine plastid, mitochondrial, and nuclear genes	18S rDNA, 26S rDNA	atpB, matK, rbcL	atp1, matR, mtSSU, mtLSU	-	-	100	9	concatenated, MP	PAUP*4.0b2	((magnoliids, (monocots, eudicots):<50):<50, basal angiosperm)	III	10.1086/431800
				-	atpB, matK, rbcL	atp1, matR,	-	-	100	5	concatenated, MP	PAUP*4.0b2	((monocots, (eudicots, magnoliids):<50):98, basal angiosperm)	I	
Saarela et al.	2007	Nature	Hydatellaceae identified as a new branch near the base of the angiosperm phylogenetic tree	-	√	-	√	-	29	-	concatenated, MP	PAUP*4.0b10	((magnoliids, (monocots, eudicots):93):100, basal angiosperm)	III	10.1038/nature05612
											concatenated, ML	PHYML 2.4.4	((magnoliids, (monocots, eudicots):95):100, basal angiosperm)	III	
Jansen et al.	2007	PNAS	Analysis of 81 genes from 64 plastid genomes resolves relationships in angiosperms and identifies genome-scale evolutionary patterns	-	√	-	-	-	64	81	concatenated, ML	GARLI 0.942	((magnoliids, (monocots, eudicots):96):100, basal angiosperm)	III	10.1073/pnas.0709121104
Moore et al.	2007	PNAS	Using plastid genome-scale data to resolve enigmatic relationships among basal angiosperms	-	√	-	-	-	45	61	concatenated, ML	GARLI	((magnoliids, (monocots, eudicots):88):100, basal angiosperm)	III	10.1073/pnas.0708072104
Burleigh et al.	2009	BMC Evolutionary Biology	Inferring phylogenies with incomplete data sets: a 5-gene, 567-taxon analysis of angiosperms	18S rDNA	atpB, rbcL	-	-	-	567	3	concatenated, ML	GARLI 0.951	((monocots, (eudicots, magnoliids):39):100, basal angiosperm)	I	10.1186/1471-2148-9-61
				18S rDNA, 26S rDNA	atpB, matK, rbcL	-	-	-	567	5	concatenated, ML	GARLI 0.951	((monocots, (eudicots, magnoliids):33):100, basal angiosperm)	I	
Soltis et al.	2009	American Journal of Botany	FLORAL VARIATION AND FLORAL GENETICS IN BASAL ANGIOSPERMS	-	-	-	IR region of the plastid genome	-	39	-	ML	-	((magnoliids, (monocots, eudicots)), basal angiosperm)	III	10.3732/ajb.0800182
Bell et al.	2010	American Journal of Botany	The age and diversification of the angiosperms re-revisited	18S rDNA	atpB, rbcL	-	-	-	567	3	concatenated, Bayesian	BEAST 1.4.8	((monocots, (eudicots, magnoliids)), basal angiosperm)	I	10.3732/ajb.0900346
Moore et al.	2010	PNAS	Phylogenetic analysis of 83 plastid genes further resolves the early diversification of eudicots	-	√	-	-	-	86	83	concatenated, ML	RAxML	((magnoliids, (monocots, eudicots):85):100, basal angiosperm)	III	10.1073/pnas.0907801107
Qiu et al.	2010	Journal of Systematics and Evolution	Angiosperm phylogeny inferred from sequences of four mitochondrial genes	-	-	atp1, matR, nad5, rps3	-	-	356	4	concatenated, ML	RAxML 7.0.4	((magnoliids, (monocots, eudicots):15):12, basal angiosperm)	III	10.1111/j.1759-6831.2010.00097.x
Soltis et al.	2011	American Journal of Botany	ANGIOSPERM PHYLOGENY: 17 GENES, 640 TAXA	18S rDNA, 26S rDNA	atpB, matK, ndhF, psbB, psbT, psbN, psbH, rbcL, rpoC2, rps16, rps4	atp1, matR, nad5, rps3	-	-	640	17	concatenated, ML	RAxML	((magnoliids, (monocots, eudicots):68):100, basal angiosperm)	III	10.3732/ajb.1000404
Moore et al.	2011	International Journal of Plant Sciences	Phylogenetic Analysis of the Plastid Inverted Repeat for 244 Species: Insights into Deeper-Level Angiosperm Relationships from a Long, Slowly Evolving Sequence Region	-	√	-	√	-	244	-	concatenated, ML	RAxML 7.2.6	((monocots, (eudicots, magnoliids):55):100, basal angiosperm)	I	10.1086/658923
Zhang et al.	2012	New Phytologist	Highly conserved low-copy nuclear genes as effective markers for phylogenetic analyses in angiosperms	SMC1, SMC2, MSH1, MLH1, MCM5	-	-	-	-	91	5	concatenated, ML	RAxML	((eudicots, (monocots, magnoliids):92):100, basal angiosperm)	II	10.1111/j.1469-8137.2012.04212.x
Xi et al.	2014	Systematic Biology	Coalescent versus Concatenation Methods and the Placement of Amborella as Sister to Water Lilies	√	-	-	-	-	45	310	coalescent	STAR	((monocots, (eudicots, magnoliids):88):100, basal angiosperm)	I	10.1093/sysbio/syu055
				-	√	-	-	-	45	45	concatenated, ML	RAxML	((magnoliids, (monocots, eudicots):54):82, basal angiosperm)	III	
Wickett et al.	2014	PNAS	Phylotranscriptomic analysis of the origin and early diversification of land plants	√	-	-	-	-	92	674	concatenated, ML	RAxML	((monocots, (eudicots, magnoliids):100):100, basal angiosperm)	I	10.1073/pnas.1323926111
									92	424	coalescent	ASTRAL	((monocots, (eudicots, magnoliids):100):100, basal angiosperm)	I	
Zeng et al.	2014	Nature Communications	Resolution of deep angiosperm phylogeny using conserved nuclear genes and estimates of early divergence times	√	-	-	-	-	61	59	concatenated, ML	RAxML	((monocots, (eudicots, magnoliids):94):100, basal angiosperm)	I	10.1038/ncomms5956
				-	√	-	-	-	86	112	concatenated, ML	RAxML	((magnoliids, (monocots, eudicots):73):100, basal angiosperm)	III	
Ruhfel et al.	2014	BMC Evolutionary Biology	From algae to angiosperms–inferring the phylogeny of green plants (Viridiplantae) from 360 plastid genomes	-	√	-	-	-	360	78	concatenated, ML	RAxML 7.3.0	((magnoliids, (monocots, eudicots):63):100, basal angiosperm)	III	10.1186/1471-2148-14-23
Wu et al.	2014	BMC Plant Biology	A precise chloroplast genome of Nelumbo nucifera (Nelumbonaceae) evaluated with Sanger, Illumina MiSeq, and PacBio RS II sequencing platforms: insight into the plastid evolution of basal eudicots	-	√	-	-	-	133	79	concatenated, ML	RAxML 7.2.8	((magnoliids, (monocots, eudicots):100):100, basal angiosperm)	III	10.1186/s12870-014-0289-0
				-	√	-	-	-	82	78			((magnoliids, (monocots, eudicots):72):100, basal angiosperm)	III	
Sun et al.	2015	Molecular Phylogenetics and Evolution	Deep phylogenetic incongruence in the angiosperm clade Rosidae	√	-	-	-	-	92	5	concatenated, ML	RAxML 7.2.8	((eudicots, (monocots, magnoliids):63):100, basal angiosperm)	II	10.1016/j.ympev.2014.11.003
				-	-	√	-	-	79	4			((eudicots, (monocots, magnoliids):54):100, basal angiosperm)	II	
Magallon et al.	2015	New Phytologist	A metacalibrated time-tree documents the early rise of flowering plant phylogenetic diversity	18S rDNA, 26S rDNA	atpB, rbcL, matK	-	-	-	792	5	concatenated, ML	RAxML 7.2.8	((magnoliids, (monocots, eudicots)), basal angiosperm)	III	10.1111/nph.13264
Sun et al.	2015	Molecular Phylogenetics and Evolution	Phylogenomic and structural analyses of 18 complete plastomes across all families of early-diverging eudicots, including an angiosperm-wide analysis of IR gene content evolution	-	√	-	-	-	97	79	concatenated, ML	RAxML 7.4.2	((magnoliids, (monocots, eudicots):65):100, basal angiosperm)	III	10.1016/j.ympev.2015.12.006

Supplementary Table 15. Summary of resequencing analysis.

Class	Province / State	Voucher No.	Resource type	Insert size (bp)	Depth	Size (Gb)	SNP	
							homozygous	heterozygous
L. chinense	Meng La (ML)	Li.ch-ML-001	Illumina, PE	150	46.00	28.22	9,212,964	1,575,766
L. chinense	Xu Yong (XY)	Li.ch-XY-001	Illumina, PE	500	26.43	16.21	6,576,024	5,813,650
L. chinense	Li Ping (LP)	Li.ch-LP-001	Illumina, PE	150	41.19	25.26	6,549,559	6,673,615
L. chinense	Sui Ning (SN)	Li.ch-SN-001	Illumina, PE	150	42.40	26.01	6,161,793	6,952,082
L. chinense	Song Tao (ST)	Li.ch-ST-001	Illumina, PE	500	26.34	16.15	6,104,629	5,992,413
L. chinense	E Xi (EX)	Li.ch-EX-001	Illumina, PE	500	27.22	16.69	6,326,851	4,742,868
L. chinense	Sang Zhi (SZ)	Li.ch-SZ-001	Illumina, PE	150	46.98	28.82	5,870,353	6,964,618
L. chinense	Liu Yang (LY)	Li.ch-LY-001	Illumina, PE	150	42.29	25.95	5,913,839	4,304,401
L. chinense	Dabie Shan (DBS)	Li.ch-DBS-001	Illumina, PE	500	34.07	20.90	5,721,144	3,320,139
L. chinense	Song Yang (SY)	Li.ch-SY-001	Illumina, PE	150	42.38	26.00	2,837,702	6,165,351
L. chinense	Huang Shan (HS)	Li.ch-HS-001	Illumina, PE	500	24.68	15.14	3,334,715	5,308,884
L. chinense	Lu Shan_1 (LS_1)	Li.ch-LS-001	Illumina, PE	500	26.16	16.04	3,048,443	5,192,742
L. chinense	Lu Shan_2 (LS_2)	Li.ch-LS-002	Illumina, PE	500	27.27	16.73	3,459,852	6,019,021
L. chinense	Wuyi Shan (WYS)	Li.ch-WYS-001	Illumina, PE	500	27.87	17.09	3,286,118	5,992,413
L. tulipifera	North Carolina (NC)	Li.tu-NC-001	Illumina, PE	500	53.72	32.95	69,018	12,012
L. tulipifera	Missouri (MO)	Li.tu-MO-001	Illumina, PE	500	53.2	32.63	69,785	10,024
L. tulipifera	Tennessee (TN)	Li.tu-TN-001	Illumina, PE	500	52.51	32.21	54,388	9,301
L. tulipifera	Georgia (GA)	Li.tu-GA-001	Illumina, PE	500	49.05	30.09	53,708	8,589
L. tulipifera	Louisiana (LA)	Li.tu-LA-001	Illumina, PE	500	57.35	35.18	86,073	16,233

L. tulipifera	Ontario (ON)	Li.tu-ON-001	Illumina, PE	350	40.9	72.6	291,180	47,119

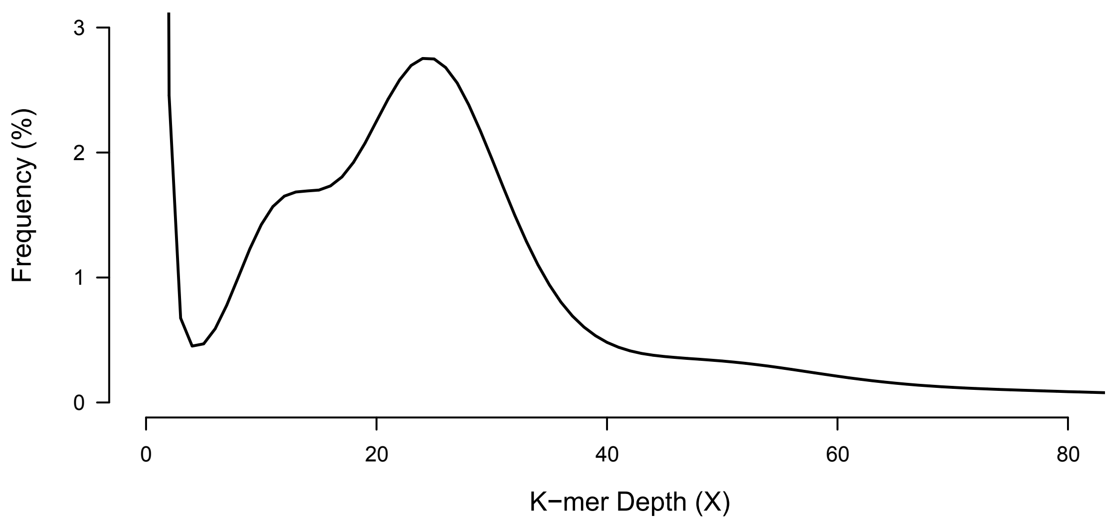

Supplementary Figure 1. k-mer frequency distribution.

The frequency and sequencing depth of 17 k-mer were plotted. Genome size was estimated using the primary peak depth and the heterozygous rate was estimated according to the second peak.

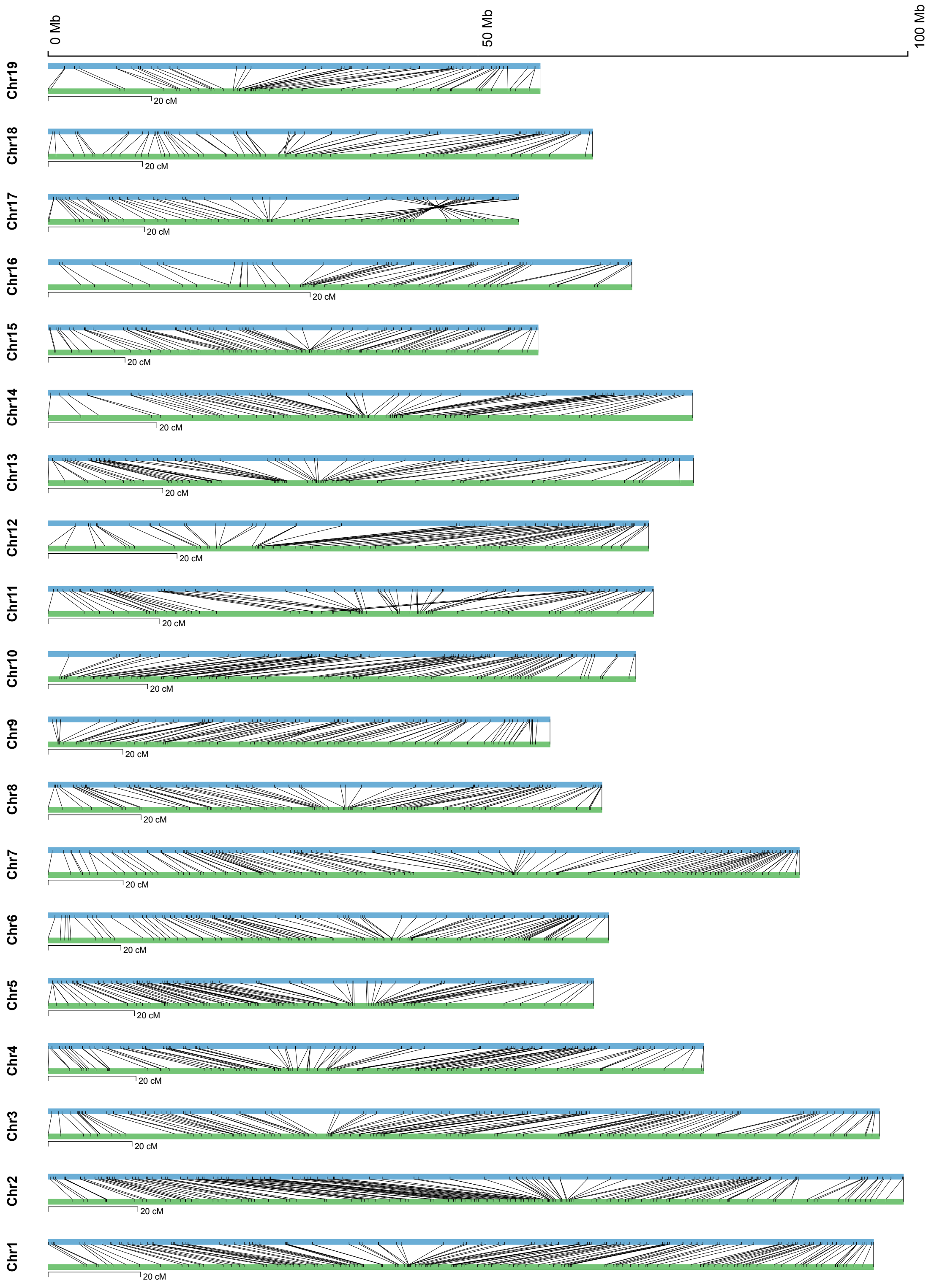

Supplementary Figure 2. Linkage map of 19 *Liriodendron* pseudo-chromosomes. The linkage map of *Liriodendron* was constructed using RAD-based SNP makers identified from 150 F1 seedlings. The green bar indicates the genetic distance with a scale of 20 cM beneath each bar, and blue bar indicates the genome sequence with a corresponding scale on the top.

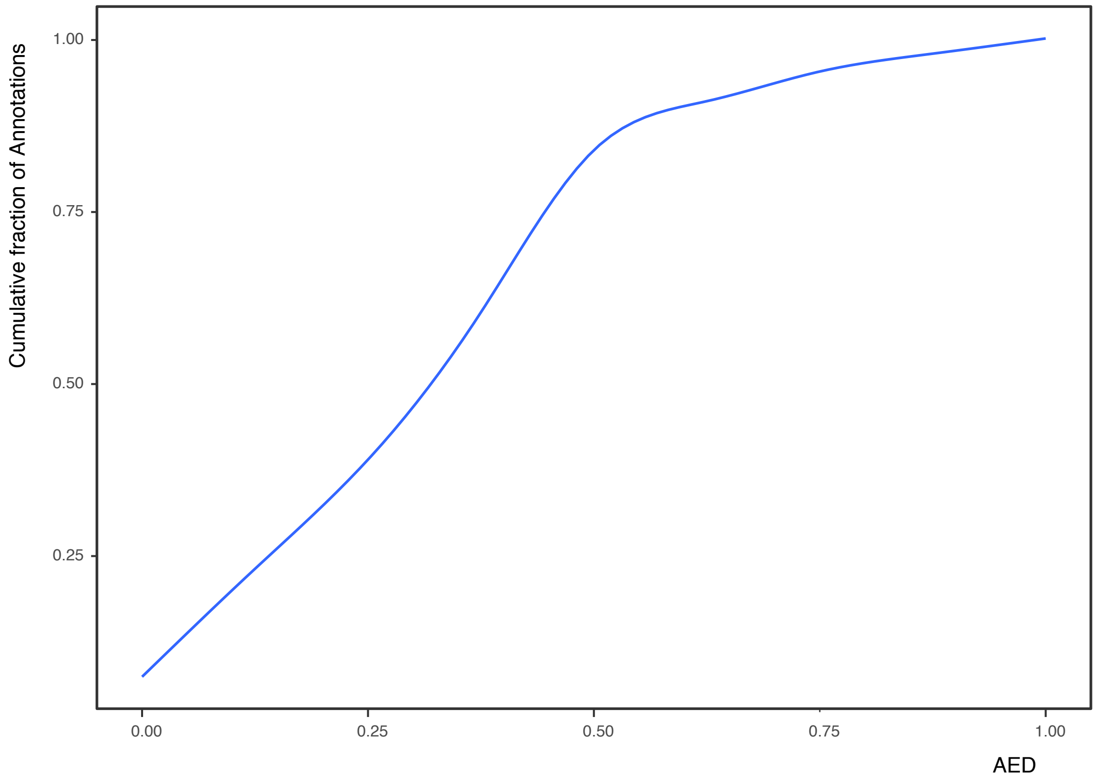

Supplementary Figure 3. Cumulative AED distributions for the *Liriodendron* genome.

Annotation Edit Distance (AED) provides a measurement for how well an annotation agrees with overlapping aligned ESTs, mRNA-seq and protein homology data. AED values range from 0 and 1, with 0 denoting perfect agreement of the annotation to aligned evidence, and 1 denoting no evidence support for the annotation.

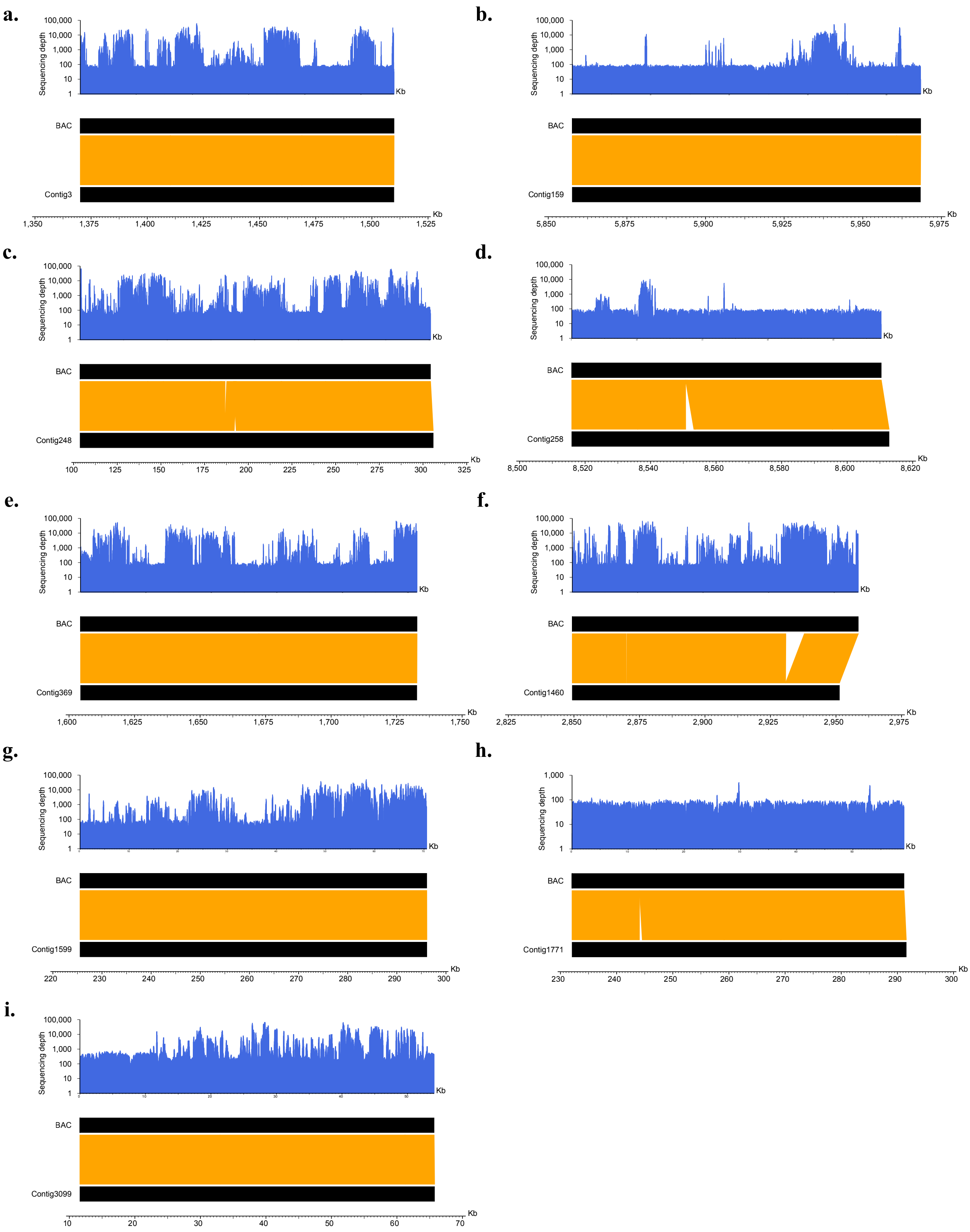

Supplementary Figure 4. Assembly quality control by assembled pooled BACs. We assembled 89 BAC sequences and mapped these BACs back to the genome assembly. Nine random alignments that indicate a low error rate are shown here. Most of the BAC sequences were covered and fewer gaps were observed in these BAC sequences than in the genome assembly.

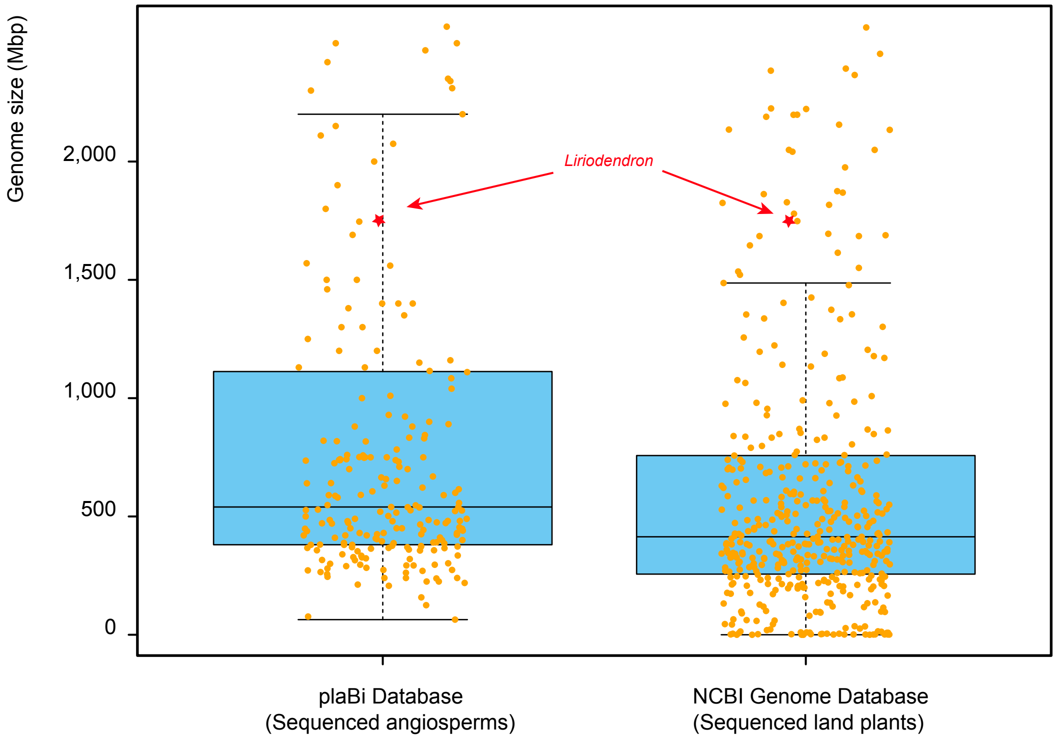

Supplementary Figure 5. Comparison of the genome size of *Liriodendron* with other sequenced plants.

The size of *Liriodendron* genome was estimated to be 1.75 Gb. Genome sizes of all sequenced angiosperms and all sequenced land plants were separately extracted from the plaBi Database (http://plabipd.de/index.ep) and the NCBI Genome Database (https://www.ncbi.nlm.nih.gov/home/genomes/). The genome size ranged from 64 Mb to 17,000 Mb with a mean value of 1,075.78 Mb in the plaBi Database and from 0.02 Mb to 27,602 Mb with a mean value of 1,060.38 Mb in the NCBI Genome Database. The genome size of *Liriodendron* was greater than those of 193 (84.65%) sequenced angiosperms and 403 (88.18%) sequenced land plants in these two databases, respectively.!

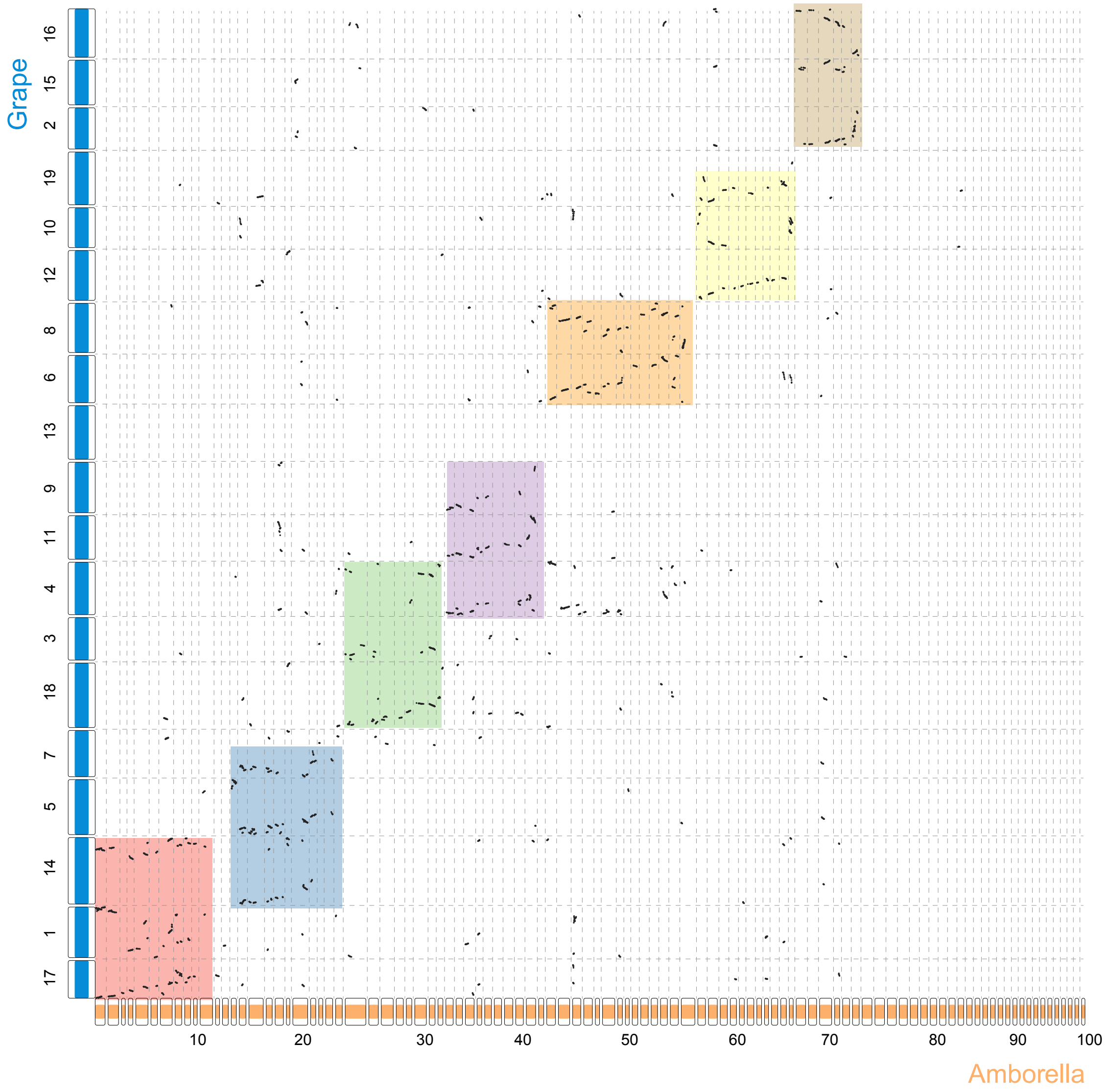

Supplementary Figure 6. Syntenic path dotplot of *Amborella* versus *Vitis*.

The y-axis represents the 19 *Vitis* chromosomes, the x-axis represents the *Amborella* scaffolds. Only the one hundred longest scaffolds were used. The *Vitis* chromosomes and *Amborella* scaffolds have been separately reordered to illustrate the 3:1 syntenic depth relationship in the comparison of *Vitis* to *Amborella* as much as possible.

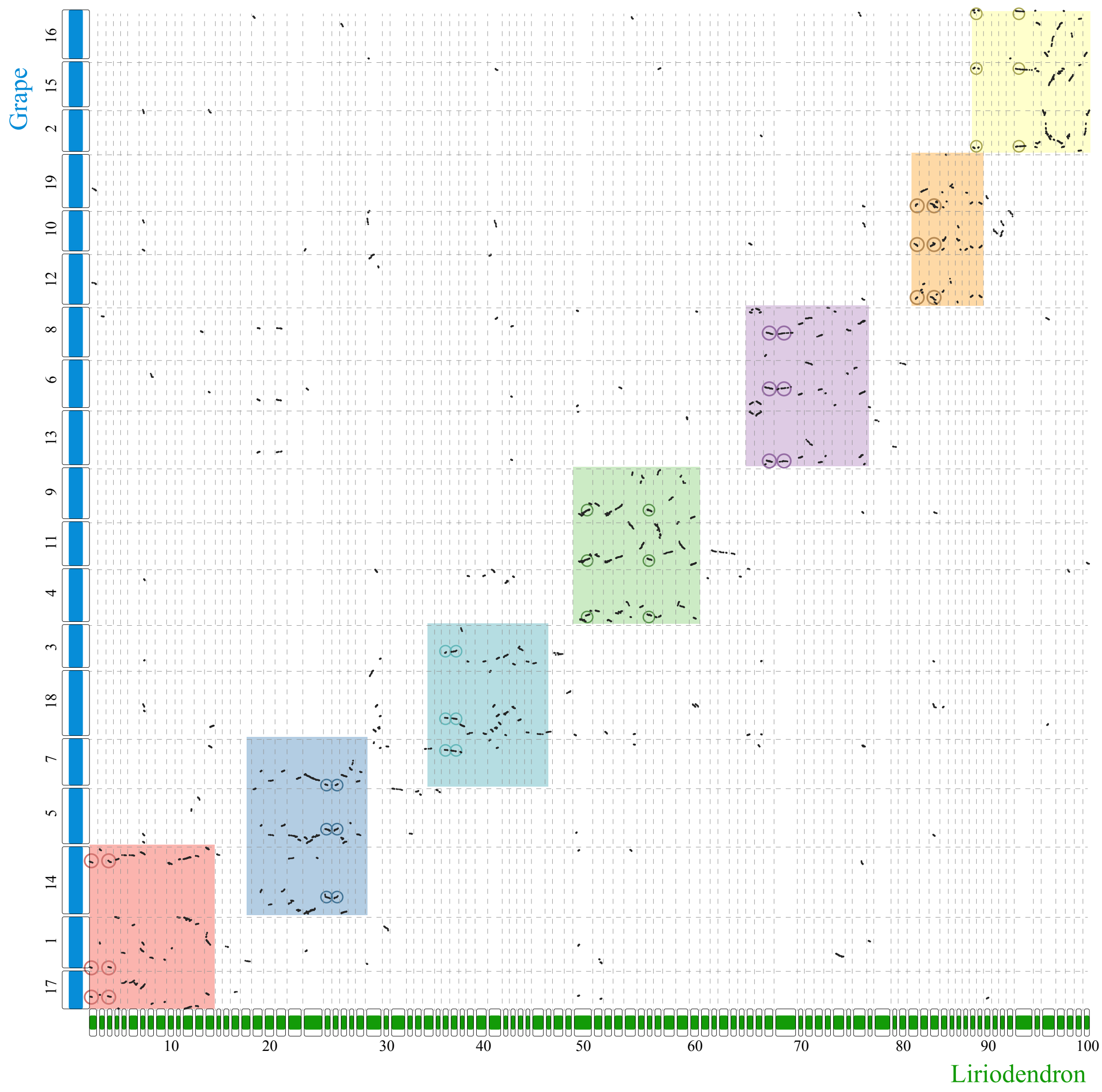

Supplementary Figure 7. Syntenic path dotplot of *Liriodendron* versus *Vitis*. The y-axis represents the 19 *Vitis* chromosomes, the x-axis represents the one hundred longest scaffolds of *Liriodendron*. The *Vitis* and *Liriodendron* scaffolds have been separately reordered to illustrate the 3:2 syntenic depth relationship in the comparison of *Vitis* to *Liriodendron* as much as possible.

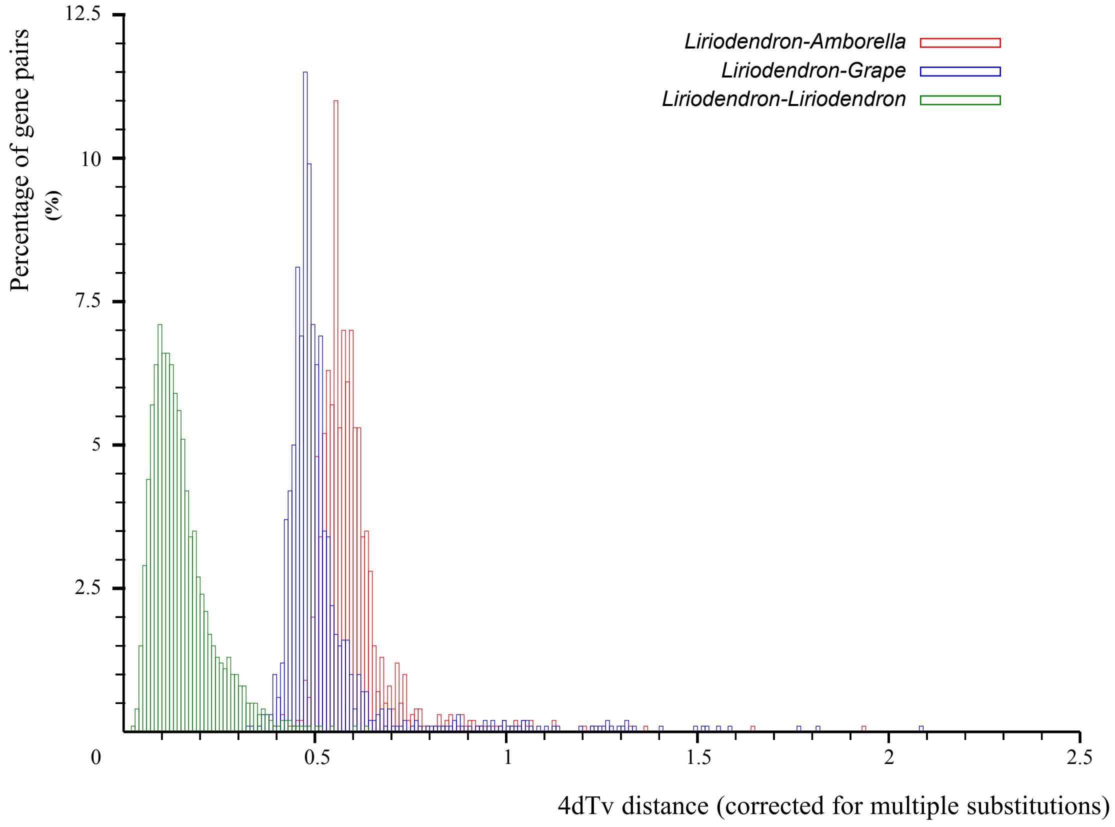

Supplementary Figure 8. 4DTV-based age distribution in *Liriodendron-Liriodendron*, *Liriodendron-Amborella* and *Liriodendron-Vitis*.

The X-axis shows the 4DTV values (with a bin of 0.05), while the Y-axis shows the number of paralogous gene pairs. The peak in *Liriodendron-Liriodendron* is 0.25 corresponding to 75~77 Mya referring to the splitting time between *Liriodendron* and *Amborella* (~180 Mya with a peak of 0.6) and grape (~154 Mya with a peak of 0.5).

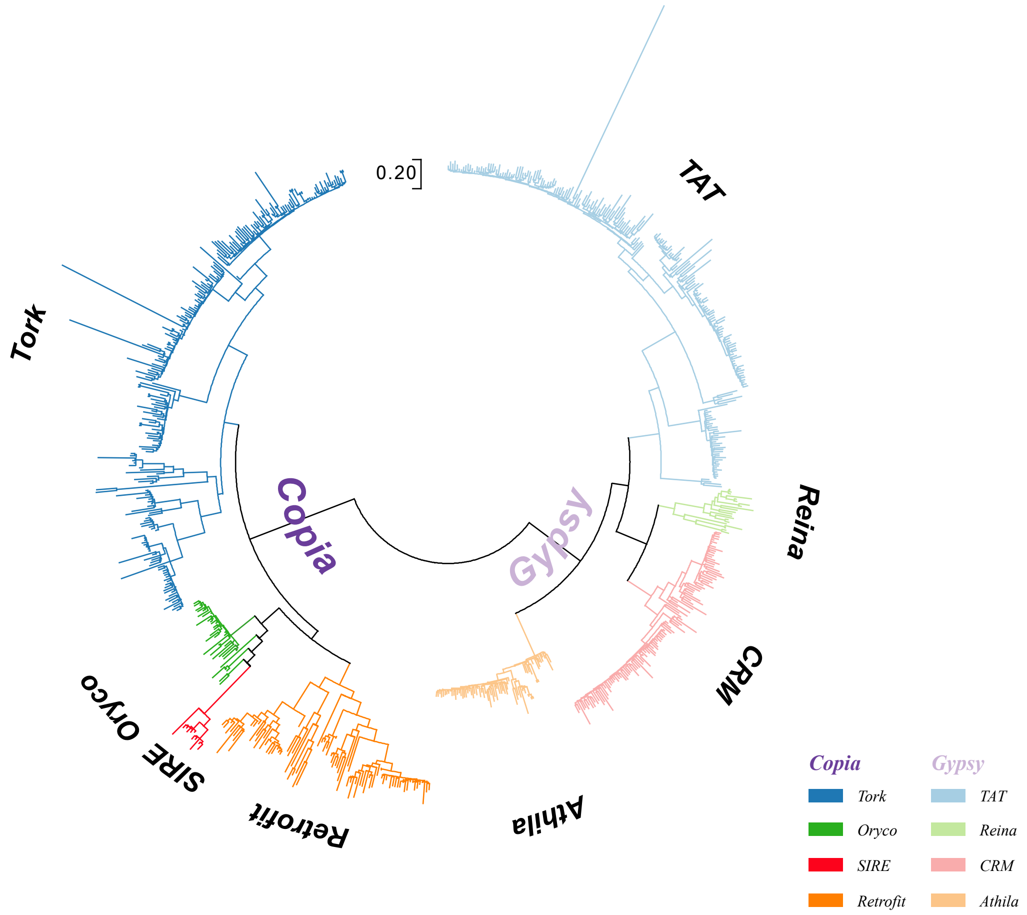

Supplementary Figure 9. Phylogenetic analysis of *Liriodendron* LTR retrotransposons.

The unrooted phylogenetic tree of *Gypsy* and *Copia* elements was constructed on the basis of the reverse-transcriptase domain sequences. The scale on the top indicates 0.2 substitution per site.

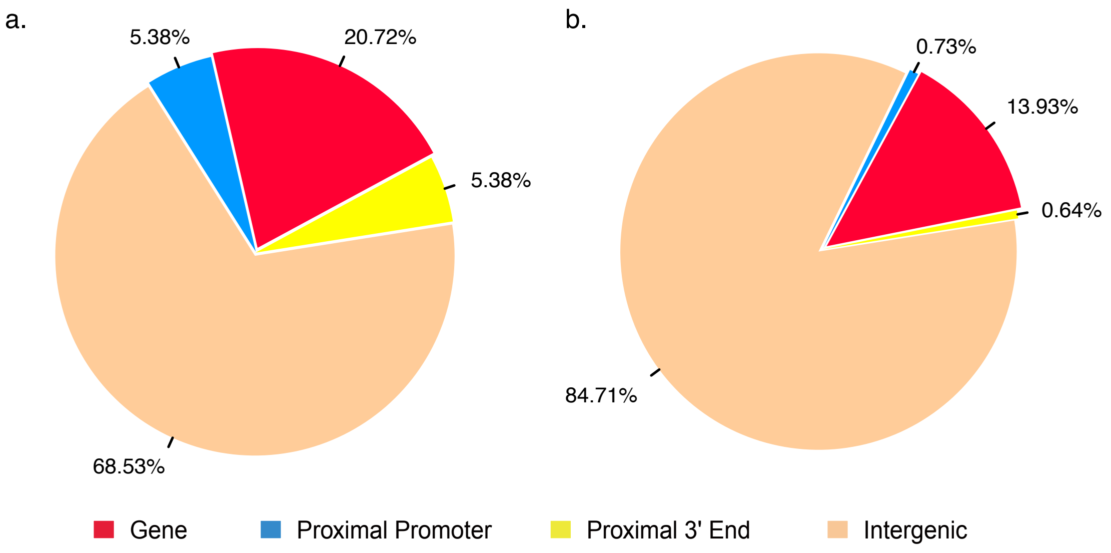

Supplementary Figure 10. An uneven TE distribution across the *Liriodendron* genome.

The pie graph demonstrates four separate *Liriodendron* genomic regions, i.e., gene (red), proximal promoter (blue), proximal 3' end (yellow) and intergenic regions (orange) accounted for the proportion of the *Liriodendron* genome (a) and TEs present in these four regions accounted for the proportion of total TEs (b). Among the TEs present in *Liriodendron* genome, 84.71% (2,834,477) located in intergenic regions, 0.73% (24,426) located in proximal promoter, 13.93% (466,111) located in genic regions and the rest 0.63% (21,081) located in proximal 3' end. If TEs are randomly distributed in *Liriodendron* genome, then the expected TE proportion of these four separate genomic regions should be the same to the proportion accounted for by these four regions in the *Liriodendron* genome, i.e., 68.52% (2,292,744), 5.38% (180,020), 20.72% (693,311) and 5.38% (180,020). Anyway, the chi-square test between the observed and expected TEs (χ^2 = 477,260, p-value = 0) showed an obvious difference.

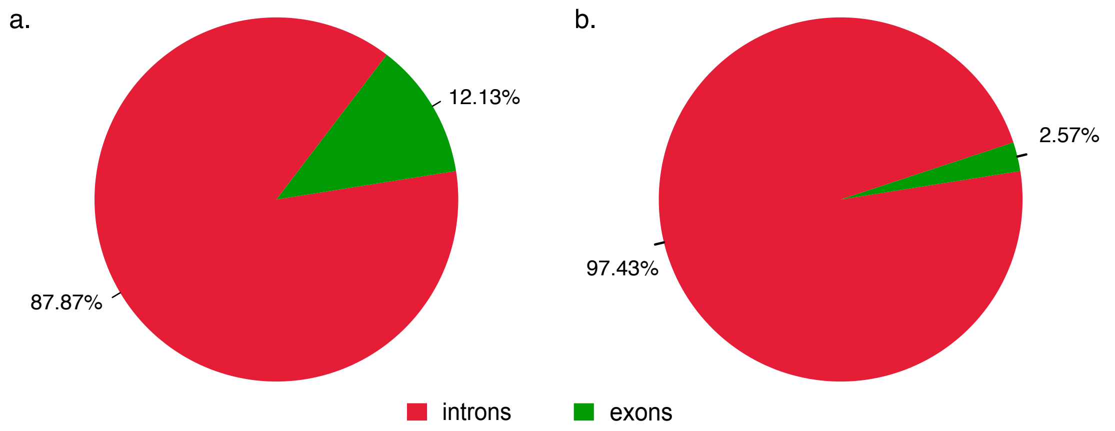

Supplementary Figure 11. TE distribution in genic regions.

The pie graph demonstrates two separate *Liriodendron* genic regions, i.e., introns (red) and exons (green) accounted for the proportion of the *Liriodendron* genic regions (a) and TEs present in these two regions accounted for the proportion of total TEs contained in genic regions (b). Among the TEs present in genic regions (with a total number of 466,111), 2.57% (11,994) located in exons and 97.43% (454,117) located in introns. If TEs are randomly distributed in genic regions, then the expected number of TEs contained in exons and introns should be 56,539.30 and 409,571.7 due to the proportion accounted for by these two regions in the *Liriodendron* genic regions. Anyway, the chi-square test between the observed and expected number (χ^2 = 39,940, p-value = 0) showed an obvious difference. The observed number of TEs located in exons is smaller than the expected number, and by contrast, the observed number of TEs located in introns is bigger than the expected number.

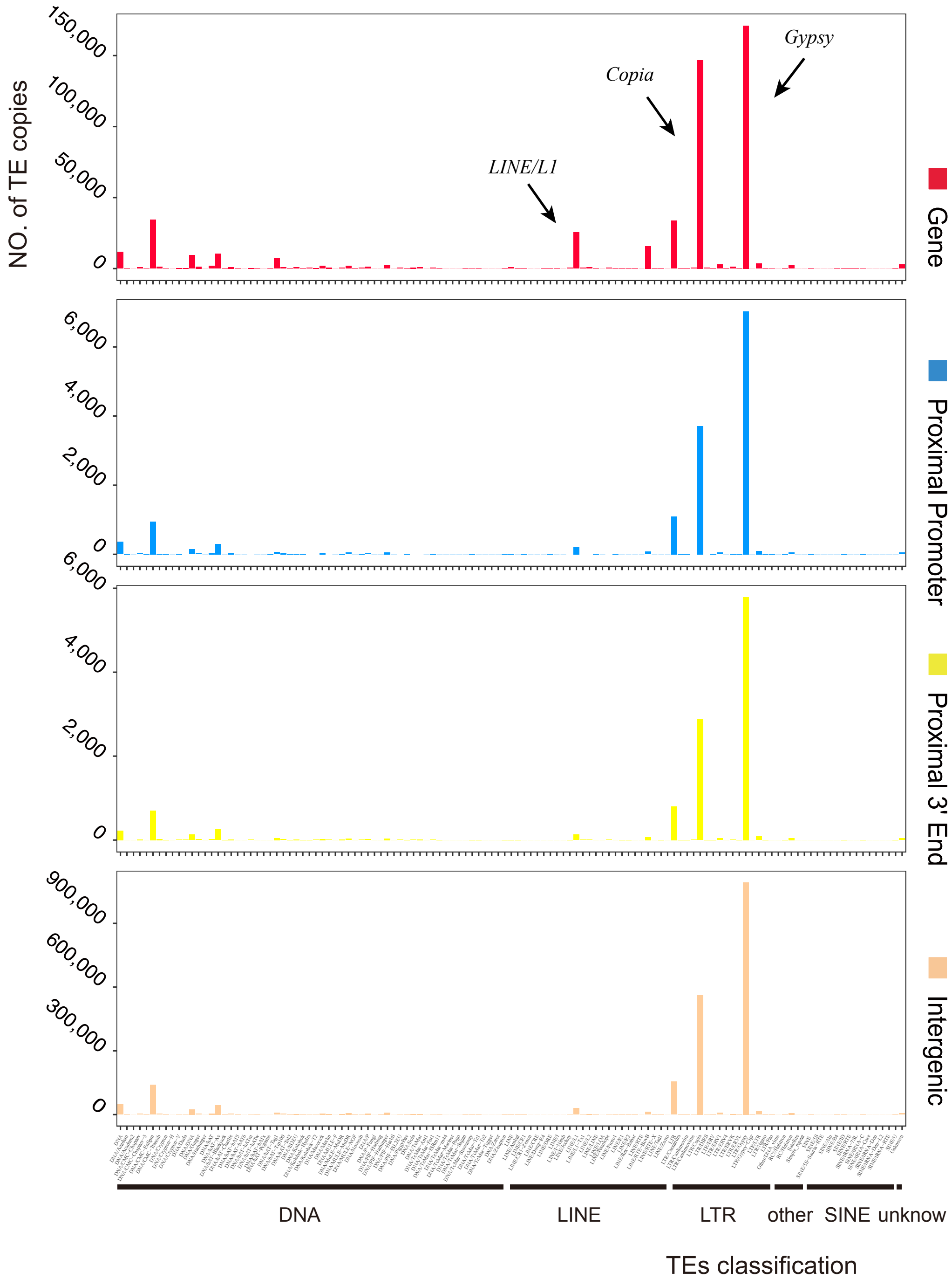

Supplementary Figure 12. TE family distribution in different *Liriodendron* genomic regions.

Within four *Liriodendron* genomic regions, TE copies of different families were separately counted and plotted. Arrows point to three TE families, which are *LINE/L1*, *Copia* and *Gypsy* from left to right.

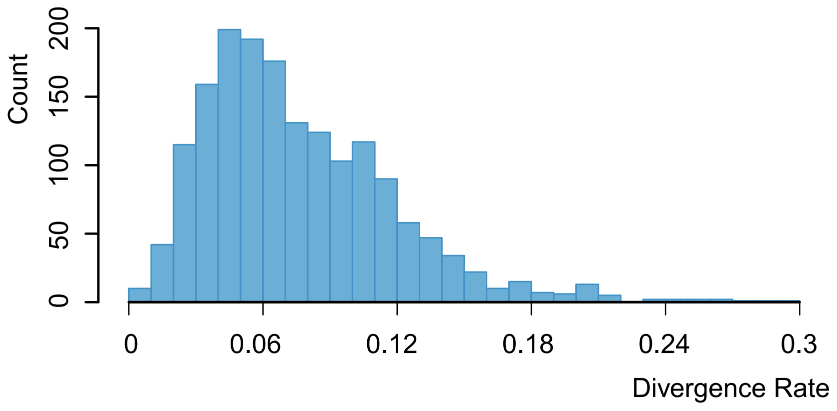

Supplementary Figure 13. LTR insertion time estimation.

Ks distributions of the complete LTR in the *L. chinense* genome are plotted by a window of 0.01.

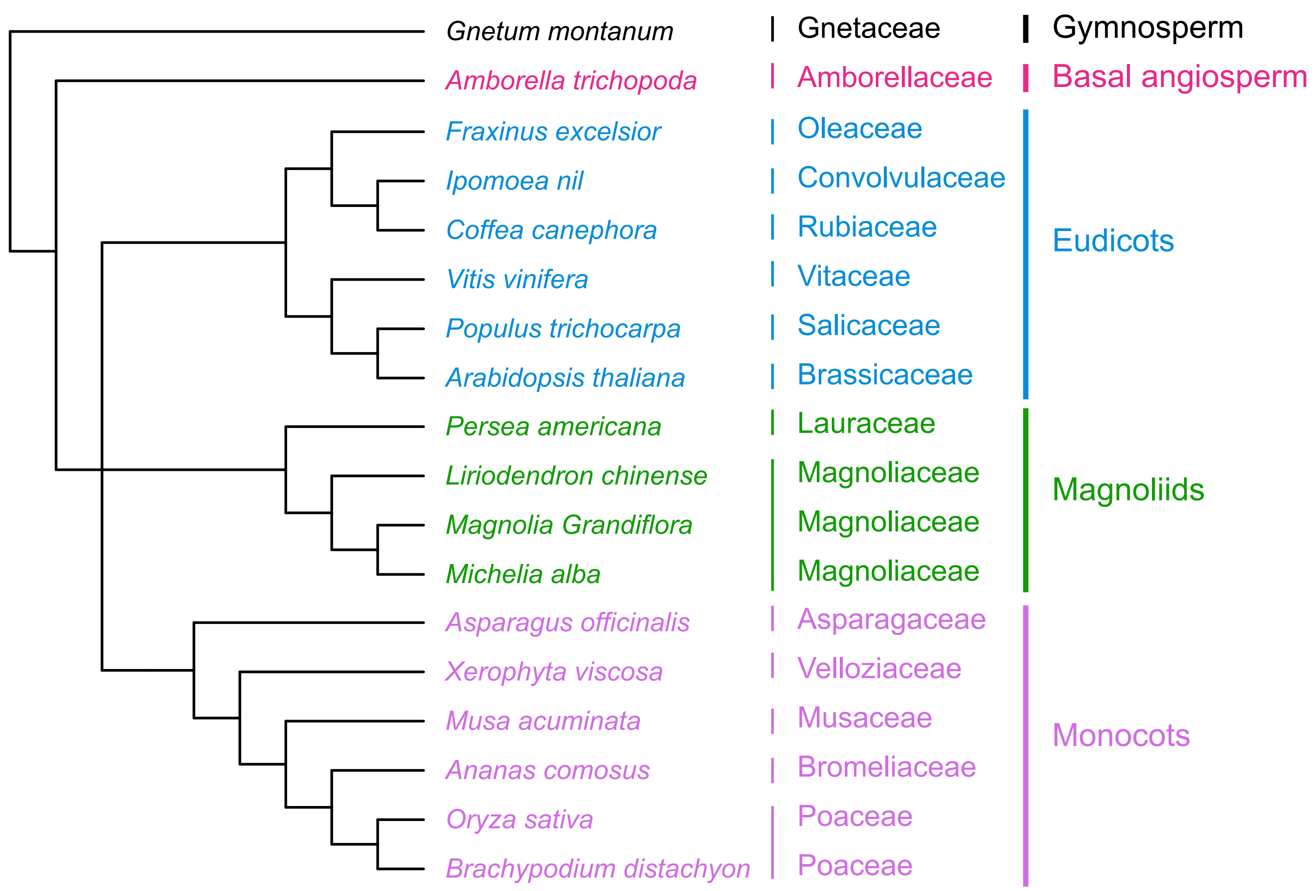

Supplementary Figure 14. A cladogram depicting established relationships of 18 representative species.

This tree was used as the reference for selecting suitable nuclear gene markers, with uncertain relationships collapsed.

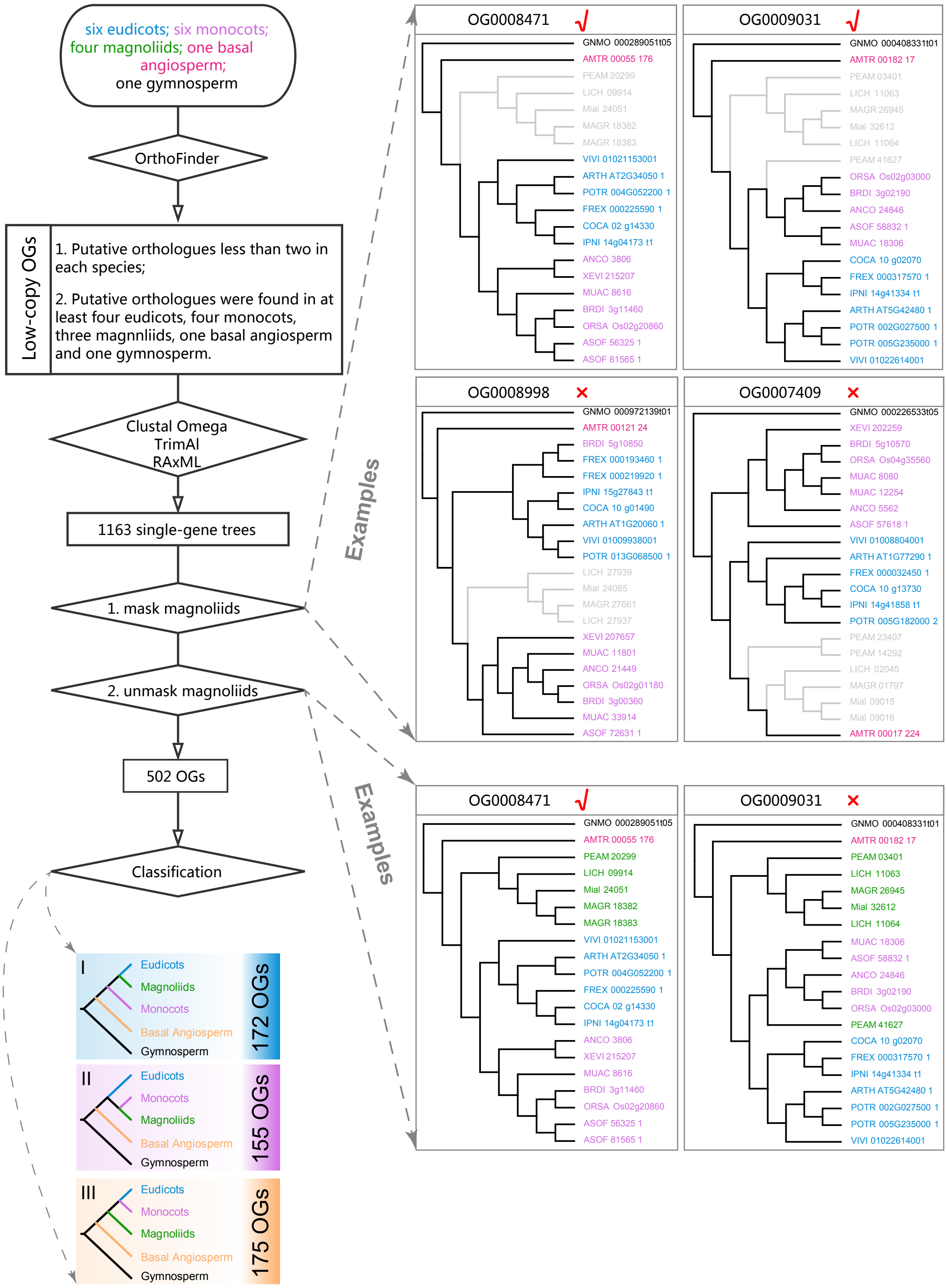

Supplementary Figure 15. The schematic flow of phylogenetic analysis and examples of single-gene trees selection.

a.

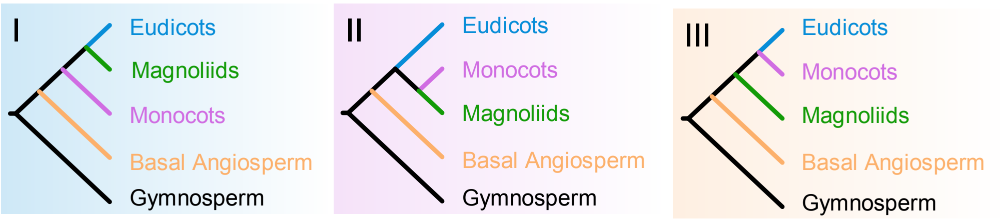

b.

Topology		Phylogenetic signal
I		166
II		167
III		169
Chisq-test	χ^2	0.0279
	p-value	0.9862

Supplementary Figure 16. The distribution of phylogenetic signal for three alternative topological hypotheses on the angiosperm lineage.

(a) Three alternative topologies are: a clade of magnoliids and eudicots as the sister group to monocots; a clade of magnoliids and monocots as the sister group to eudicots; magnoliids as the sister group to the clade of eudicots and monocots. (b) Distribution of genes supporting each of three alternative hypotheses for the 502 low-copy OG dataset.

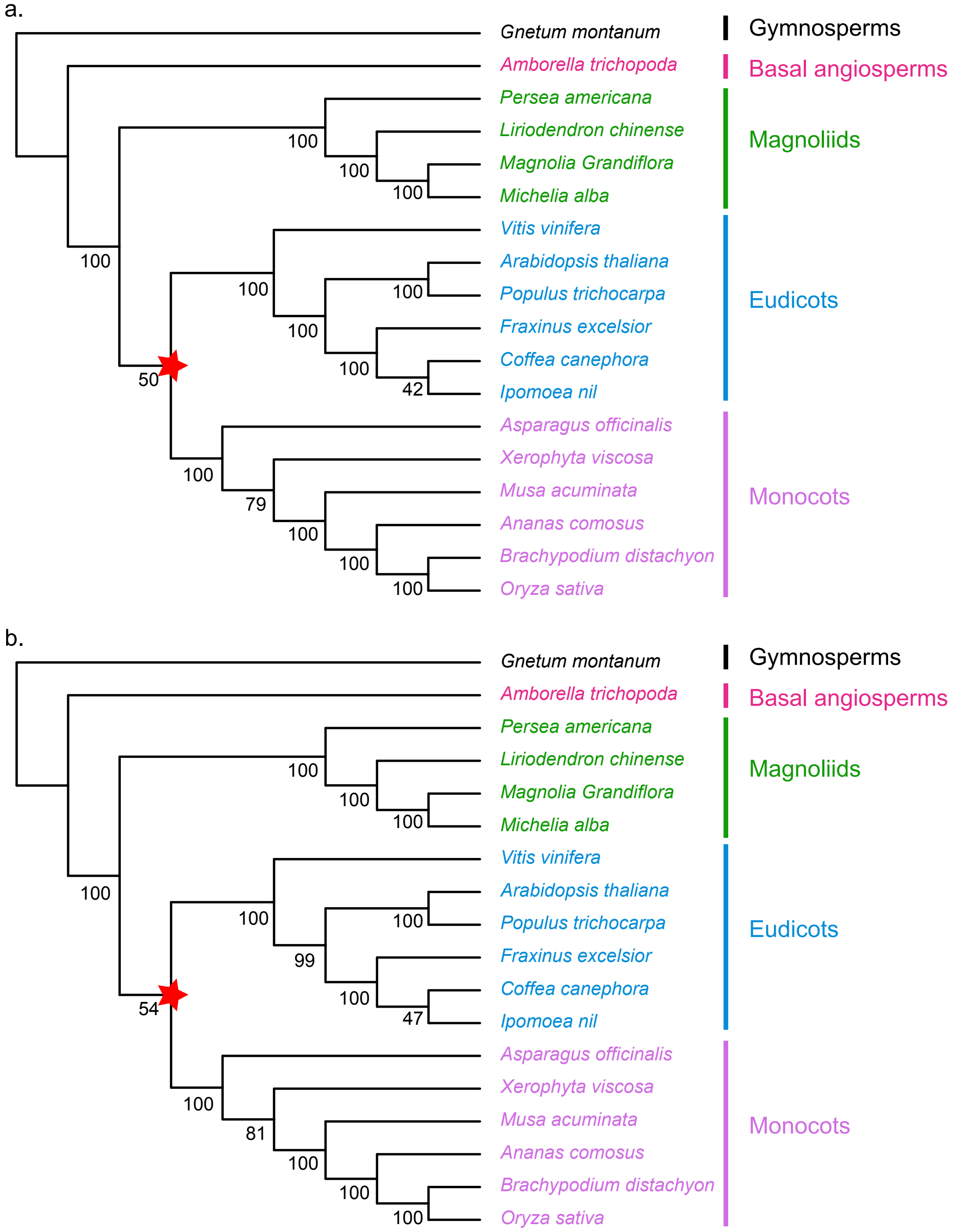

Supplementary Figure 17. Phylogenetic trees based on the 502-OG and 481-OG datasets of 18 land plant species.

(a) Protein sequences of 502 low-copy OGs were separately aligned, trimmed and used to infer single-gene phylogenies. Then, only the orthologue gene with the shortest branch length in each species was retained in each OGs for following species tree estimation using ASTRAL. (b) OGs with outlier ΔGLS values were excluded and the remaining 481 OGs were used to estimate the species tree using ASTRAL. Numbers associated with nodes are bootstrap values.

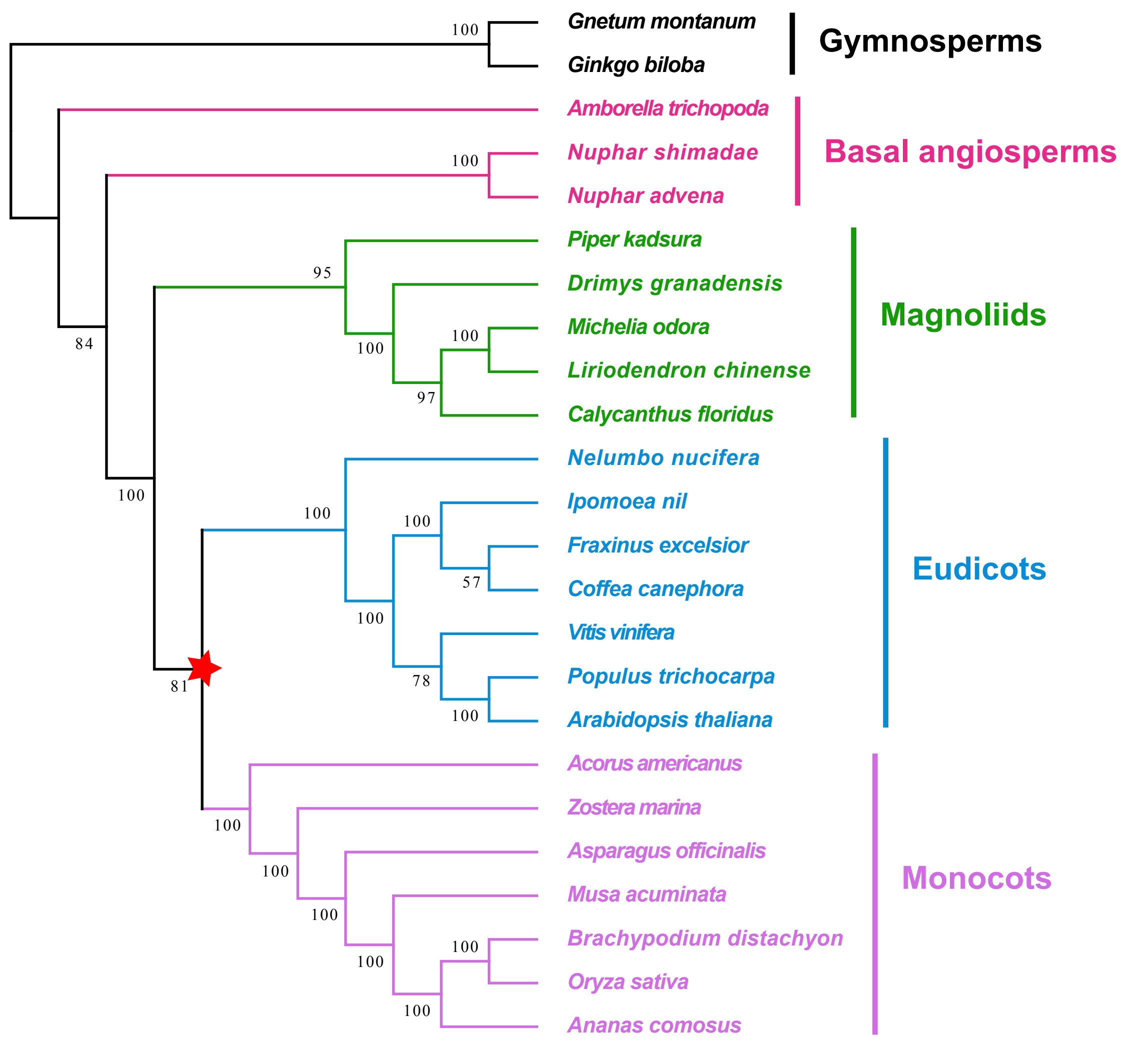

Supplementary Figure 18. The phylogenetic tree based on 78 chloroplast genes from 24 species.

The phylogenetic tree was constructed from 78 concatenated chloroplast gene sequences that were shared among 24 plant species using the ML method. Numbers associated with nodes are bootstrap values.

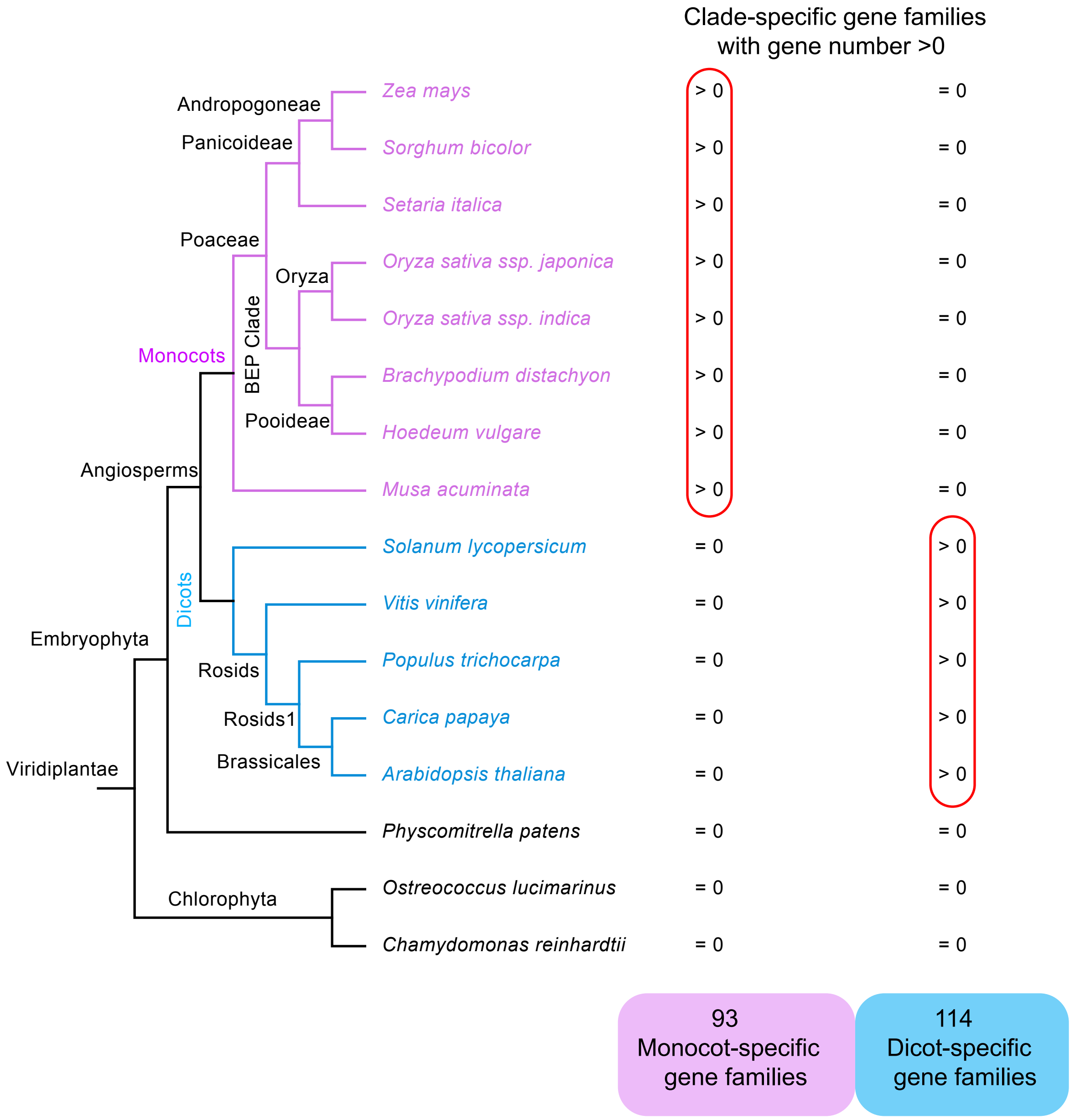

Supplementary Figure 19. Monocot- and dicot-specific gene family selection.

We found monocot- and dicot-specific gene families based on phylogenetic profiles in the Monocots PLAZA 3.0 database. We manually selected all the species that came from the target clade, i.e., monocots or dicots, for identifying clade-specific gene families with all species included and setting the gene number =0 within nontarget clade species. Finally, we separately obtained 93 monocot- and 114 dicot-specific gene families.

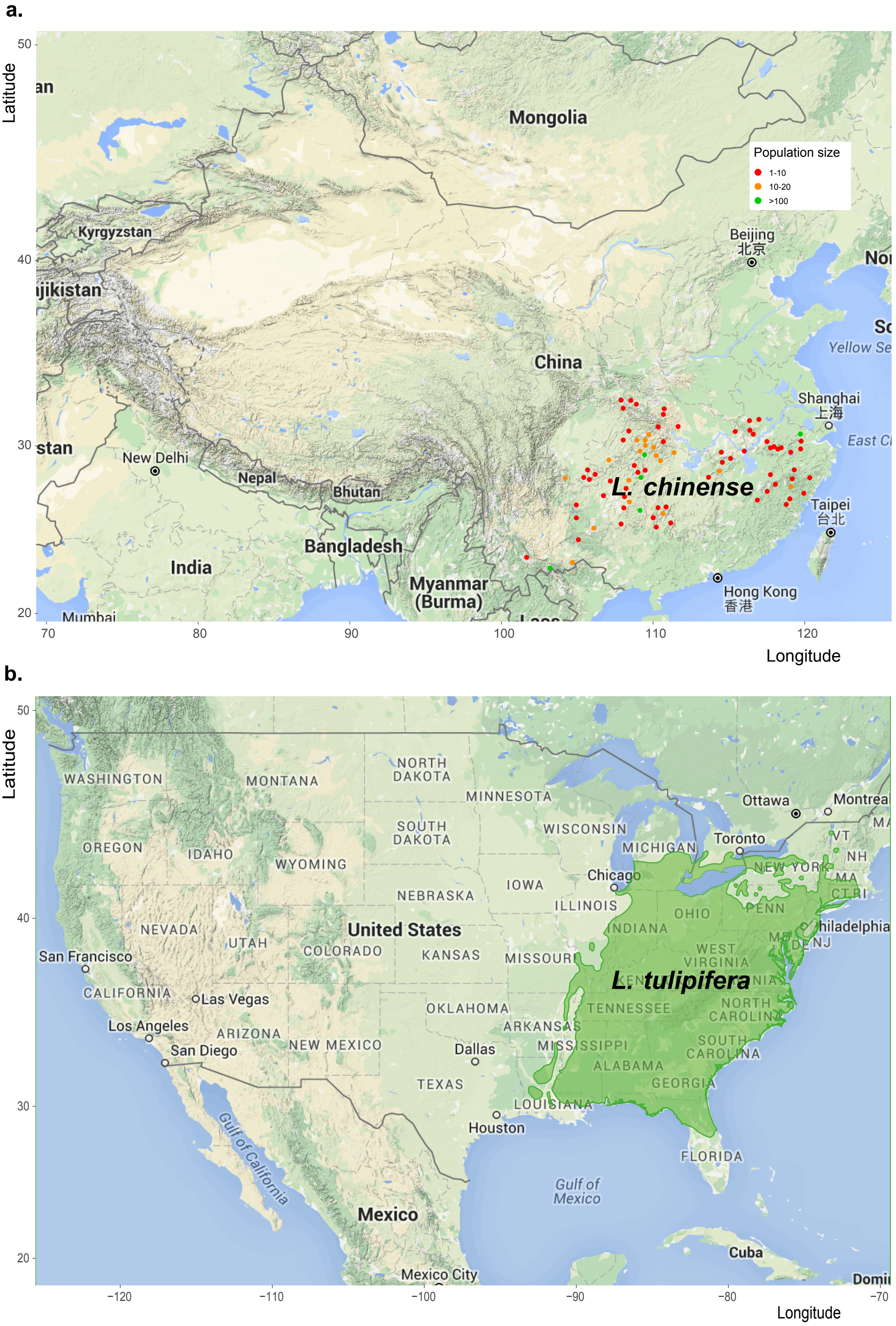

Supplementary Figure 20. Natural distribution of the two *Liriodendron* species.

The natural distribution maps of *L. chinense* (a) and *L. tulipfera* (b) were separately plotted. The *L. chinense* natural distribution data was obtained from Hao *et al.* (1995) and the *L. tuplifera* natural distribution data were downloaded from the Geosciences and Environmental Change Science Center (GECSE; http://esp.cr.usgs.gov/) database.

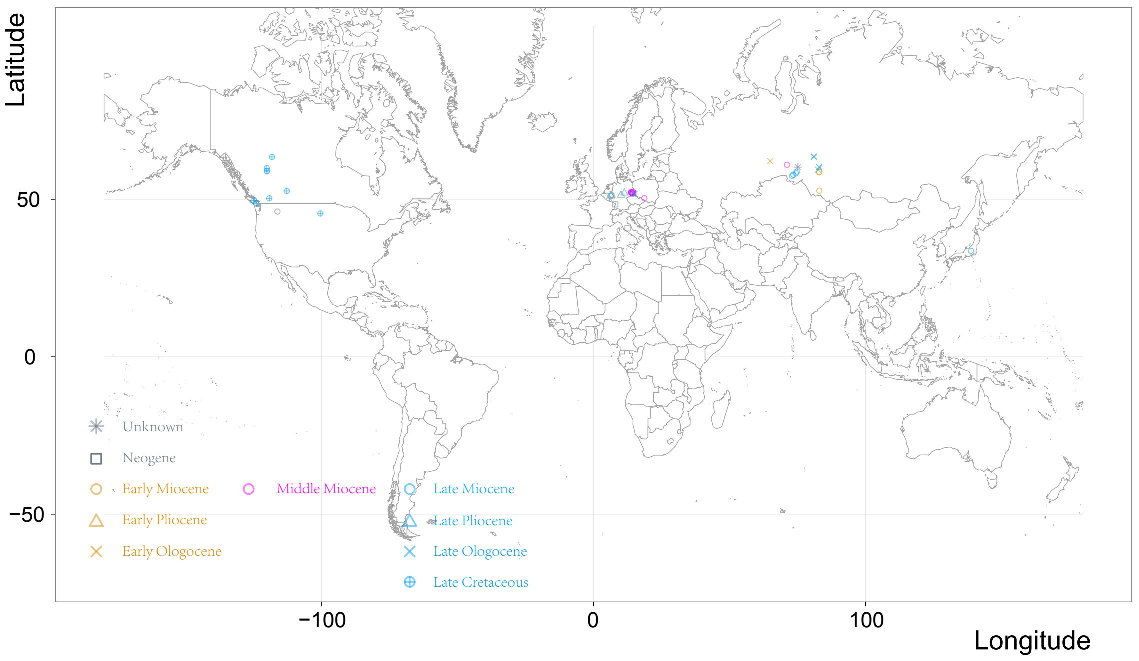

Supplementary Figure 21. Distribution of extinct *Liriodendron* species in high-latitude regions before the Late Tertiary.

Different colors and shape symbols represented different geological ages which were inferred from the fossils. The data were downloaded from the Fossilworks database.

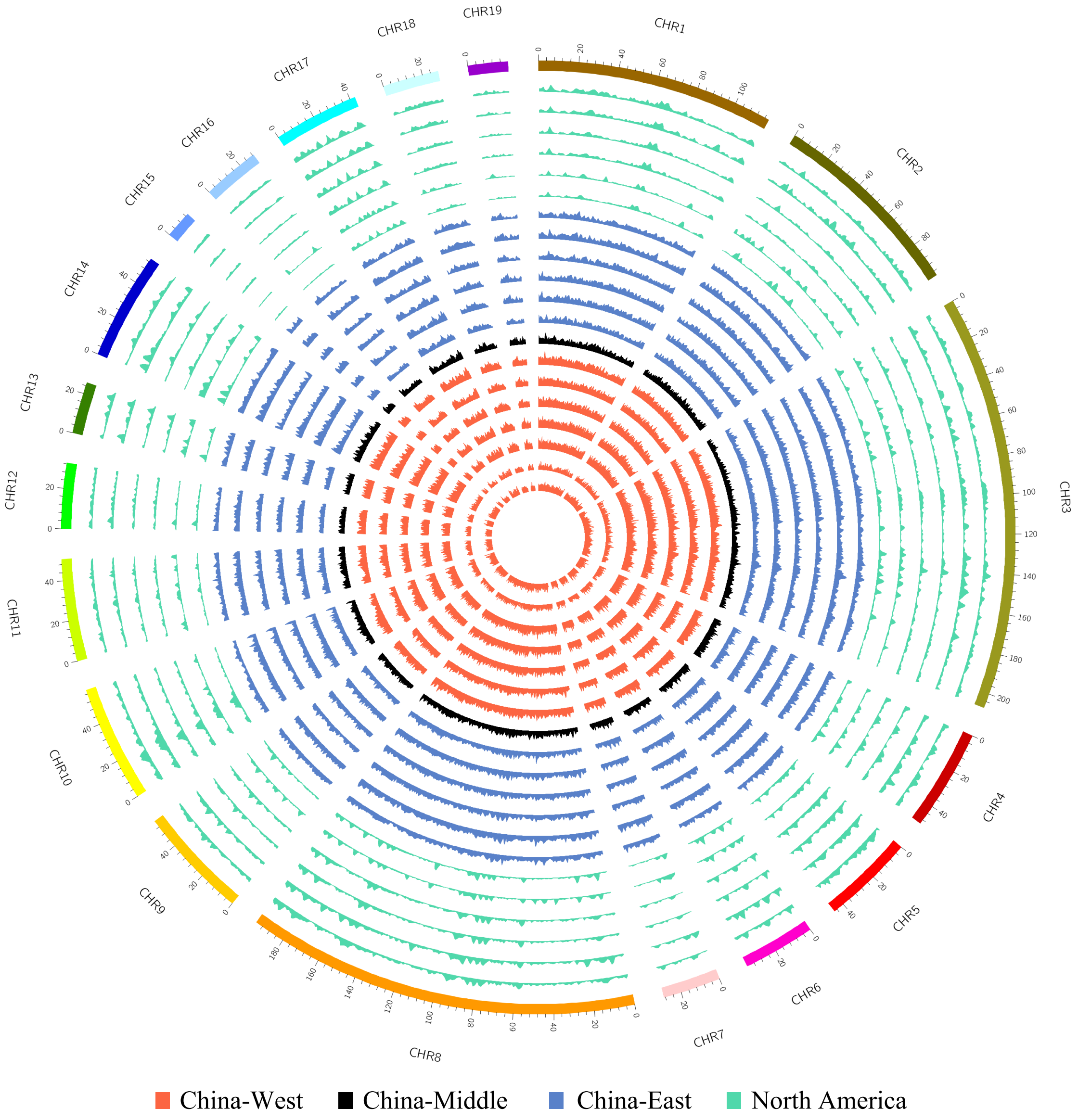

Supplementary Figure 22. Overview of SNP distribution among 20 resequenced individuals.

The 20 inner tracks depict SNP frequency distributions for 1-Mb non-overlapping windows in the seven *L. chinense* that came from Western China, one *L. chinense* that came from Central China, six *L. chinense* that came from Eastern China, and six *L. tuplifera* that came from North America.

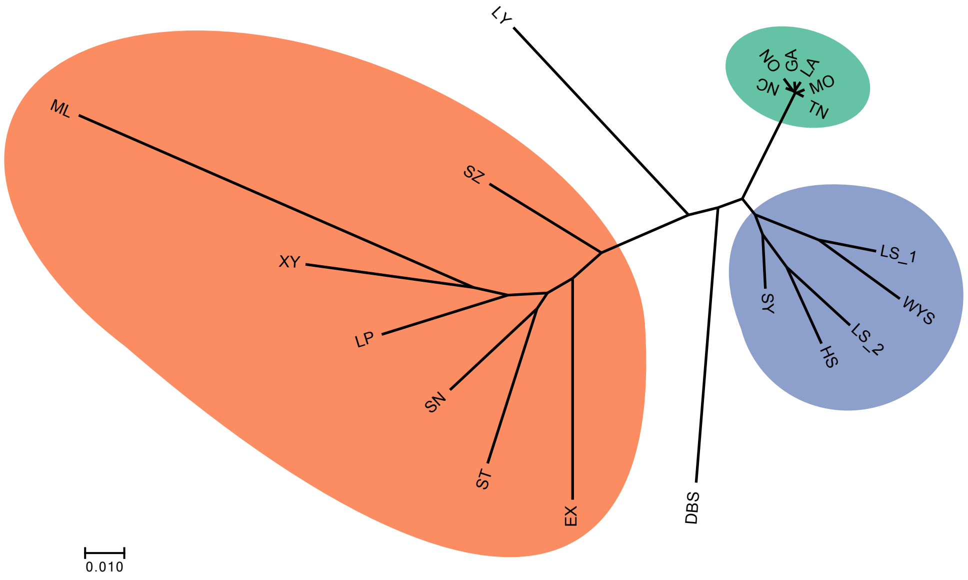

Supplementary Figure 23. A SNP tree reconstructed using RAxML.

The ML tree of all accessions constructed from whole-genome SNPs. Accessions coming from the same geographic areas are grouped together and colored corresponding to colors used in Figure 3.

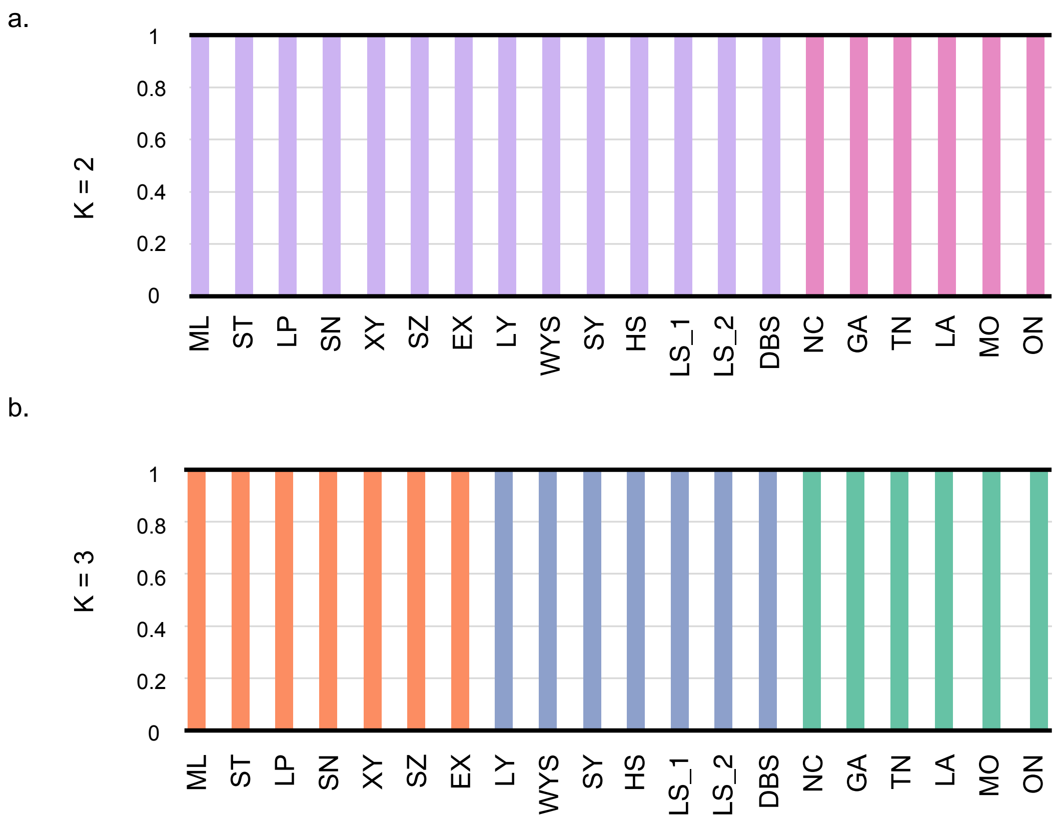

Supplementary Figure 24. Population structure analysis.

Varying the number of presumed ancestral populations (K) showed that 20 *Liriodendron* resequenced individuals were divided into two groups, *L. chinense* and *L. tulipifera*, when K = 2, and three distinct groups when K = 3 (b).

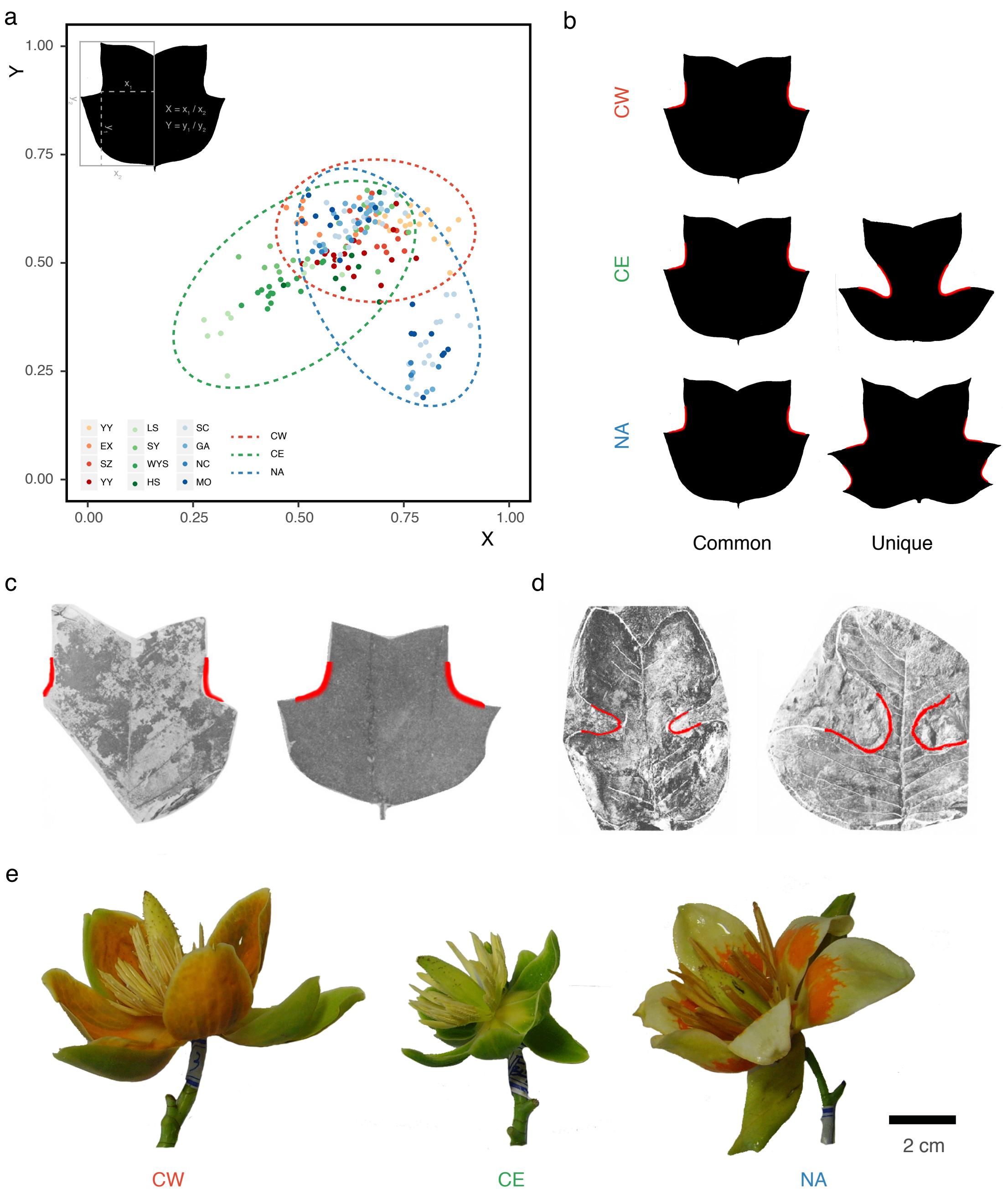

Supplementary Figure 25. Phenotypic Analysis.

(a) The relative positions of the lateral sinus located in the left half of the leaf were plotted. The X-axis represents the ratio of the vertical distance from the lateral sinus to the primary vein (x_1) to the vertical distance from the lateral lobe to the primary vein (x_2). The Y-axis represents the ratio of the vertical distance from the lateral sinus to the leaf blade base (y_1) to the vertical distance from the apical lobe to the leaf blade base (y_2). (b) The representative leaf shapes of three groups were plotted respectively. (c) and (d) were the leaf shapes of two extinct *Liriodendron* species, *L. hesperia* and *L. giganteum*, respectively. (e) The representative mature floral organs of three *Liriodendron* groups. The experiment was repeated independently at least three times with similar results.

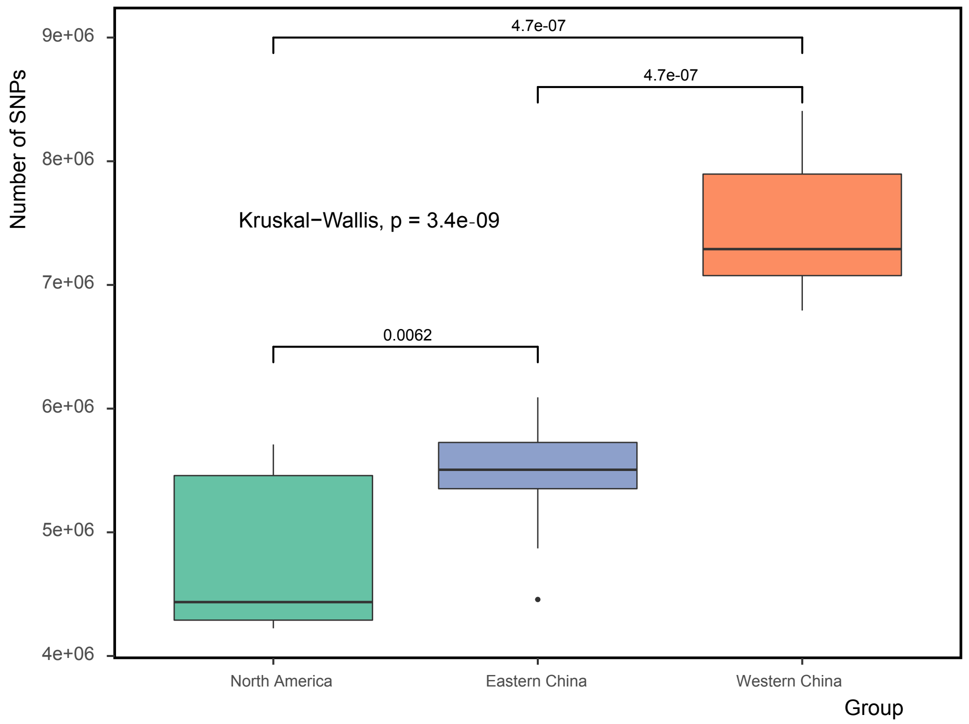

Supplementary Figure 26. Individual differences within three *Liriodendron* groups.

The X-axis represents the three *Liriodendron* groups supported by the SNP tree, PCA and structure analysis. Six, six, and seven individuals were separately included within these three group from left to right. The Y-axis represents inter-individual SNPs within three groups. The number of inter-individual SNP ranged from 4,224,002 to 5,710,354 with a mean value of 4,766,498 in the North America group, from 4,456,851 to 6,091,489 with a mean value of 5,485,145 in the Eastern China group, and from 6,793,165 to 8,407,025 with a mean value of 7,446,489 in the Western China group.

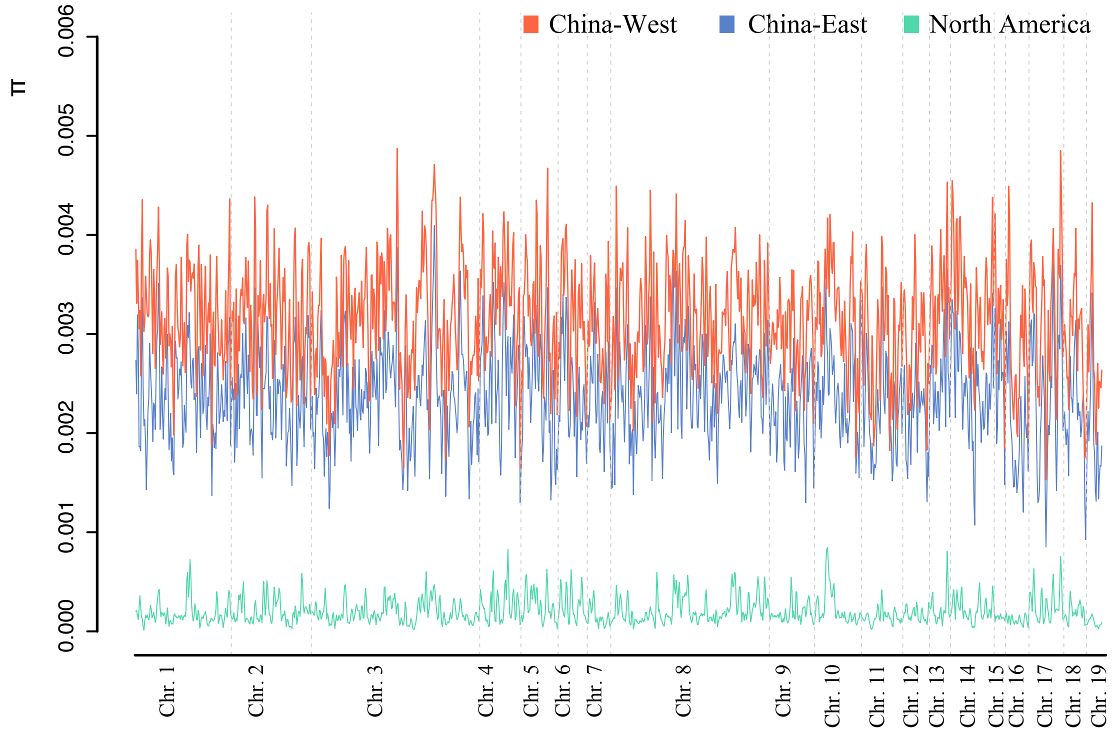

Supplementary Figure 27. Distribution of π along 20 *Liriodendron* chromosomes.

Distributions of π along 20 *Liriodendron* chromosomes among CW, CE and NA groups, respectively are plotted. These values are calculated in a 2-Mb sliding window with a 1-Mb step.
