# Supplementary material for: Liriodendron genome sheds light on angiosperm phylogeny and species–pair differentiation
Source: Nat Plants. 2018 Dec 17;5(1):18–25. doi: 10.1038/s41477-018-0323-6 (PMC6784881; doi:10.1038/s41477-018-0323-6)
Supplement: Supplementary file 2 — Reporting Summary [file 41477_2018_323_MOESM2_ESM.pdf]

nature research

Corresponding author(s): Jisen Shi

Reporting Summary

Nature Research wishes to improve the reproducibility of the work that we publish. This form provides structure for consistency and transparency in reporting. For further information on Nature Research policies, see Authors & Referees and the Editorial Policy Checklist.

Statistical parameters

When statistical analyses are reported, confirm that the following items are present in the relevant location (e.g. figure legend, table legend, main text, or Methods section).

n/a	Confirmed	
☐	☒	The exact sample size (*n*) for each experimental group/condition, given as a discrete number and unit of measurement
☐	☒	An indication of whether measurements were taken from distinct samples or whether the same sample was measured repeatedly
☐	☒	The statistical test(s) used AND whether they are one- or two-sided<br>*Only common tests should be described solely by name; describe more complex techniques in the Methods section.*
☒	☐	A description of all covariates tested
☒	☐	A description of any assumptions or corrections, such as tests of normality and adjustment for multiple comparisons
☒	☐	A full description of the statistics including central tendency (e.g. means) or other basic estimates (e.g. regression coefficient) AND variation (e.g. standard deviation) or associated estimates of uncertainty (e.g. confidence intervals)
☐	☒	For null hypothesis testing, the test statistic (e.g. *F*, *t*, *r*) with confidence intervals, effect sizes, degrees of freedom and *P* value noted<br>*Give P values as exact values whenever suitable.*
☒	☐	For Bayesian analysis, information on the choice of priors and Markov chain Monte Carlo settings
☒	☐	For hierarchical and complex designs, identification of the appropriate level for tests and full reporting of outcomes
☒	☐	Estimates of effect sizes (e.g. Cohen's *d*, Pearson's *r*), indicating how they were calculated
☐	☒	Clearly defined error bars<br>*State explicitly what error bars represent (e.g. SD, SE, CI)*

Our web collection on statistics for biologists may be useful.

Software and code

Policy information about availability of computer code

Data collection	No software was used to collect the data.
Data analysis	We used lots of software for data analysis in this paper. FALCON, SMRT Link v5.0.0, BWA-mem v0.7.17, Pilon v1.21 and PBJelly v15.8.24 were used in genome assembly. JoinMap v4.0 was used in linkage map construction. SOAPdenovo v2.04, BLASTN v2.3.0, Trinity v2.4.0 and BLAT v35 were used in genome assessment. Tandem Repeats Finder v4.04, RepeatMasker, RepeatModeler v1.0.11, TBLASTN v2.3.0, MAKER v2.31.10, BLASTP v2.3.0, InterProScan, tRNAscan-SE v1.3.1, BLASTN v2.3.0 and INFERNAL v1.1.2 were used in genome annotation. BLASTP v2.3.0, MCscan v0.8, MUSCLE, PAML v4.8, OrthoMCL v5, PRANK and PhyML v3.0 were used in whole genome duplication identification. OrthoFinder v2.2.3, Clustal Omega v1.2.4, TrimAl v1.2, RAxML v8.2.11, ASTRAL v5.6.1, PAML MCMCTREE, BLASTP v2.3.0 and Café v4.0.1 were used in phylogenetic analysis. BWA v0.7.17, SAMtools v1.3.1, GATK v3.2.2, SNPEFF, TreeBeST v1.9.2, RAxML v8.2.11, PLINK v1.07, FRAPPE v1.1, ADMIXTURE v1.3.0, EIGENSOFT v3.2 and R were used in population structure analysis.

For manuscripts utilizing custom algorithms or software that are central to the research but not yet described in published literature, software must be made available to editors/reviewers upon request. We strongly encourage code deposition in a community repository (e.g. GitHub). See the Nature Research guidelines for submitting code & software for further information.

Data

Policy information about availability of data

All manuscripts must include a data availability statement. This statement should provide the following information, where applicable:

- Accession codes, unique identifiers, or web links for publicly available datasets
- A list of figures that have associated raw data
- A description of any restrictions on data availability

Raw reads and genome assembly have been deposited as a BioProject under accession PRJNA418360. Resequencing data have been deposited as a BioProject under accession PRJNA418361.

Field-specific reporting

Please select the best fit for your research. If you are not sure, read the appropriate sections before making your selection.

☒ Life sciences ☐ Behavioural & social sciences

For a reference copy of the document with all sections, see nature.com/authors/policies/ReportingSummary-flat.pdf

Life sciences

Study design

All studies must disclose on these points even when the disclosure is negative.

Sample size	The size of F1 progenies, i.e., 150 individuals, in Liriodendron is in the standard for linkage map construction.
Data exclusions	The reads with low quality are more likely to contain errors, which might complicate the following assembly process, and were excluded. Detailed criteria were provided in Supplementary Note 1.3.
Replication	The phenotypic characteristics of Liriodendron chinense were identified independently more than ten times.
Randomization	All samples were treated the same and no randomization was performed.
Blinding	The Liriodendron genome were sequenced and assembled with no blinding. All seqeuncing data came from the same adult tree; therefore blinding is not relevant to these analyses.

Materials & experimental systems

Policy information about availability of materials

n/a	Involved in the study
☐	☒ Unique materials
☒	☐ Antibodies
☒	☐ Eukaryotic cell lines
☒	☐ Research animals
☒	☐ Human research participants

Unique materials

Obtaining unique materials	All Liriodendron individuals used in this study were planted in a forest farm of Nanjing Forestry University, China. Please contact authors for further information.

Method-specific reporting

n/a	Involved in the study
☒	☐ ChIP-seq
☐	☒ Flow cytometry
☒	☐ Magnetic resonance imaging

Flow Cytometry

Plots

Confirm that:

- [ ] The axis labels state the marker and fluorochrome used (e.g. CD4-FITC).
- [ ] The axis scales are clearly visible. Include numbers along axes only for bottom left plot of group (a 'group' is an analysis of identical markers).
- [ ] All plots are contour plots with outliers or pseudocolor plots.
- [ ] A numerical value for number of cells or percentage (with statistics) is provided.

Methodology

Sample preparation	Yong leaves of this Liriodendron individual used for the whole genome sequencing together with young leaves of Vinca major were first "chopped" with a sharp razor blade in 500µl Extraction Buffer (ice-cold), in a plastic petri disc. After 30-60 seconds of incubation, 2.0 ml Staining Buffer is added. This buffer contains Propidium Iodide (PI) as fluorescent dye and RNA-se. To the buffer is also added 0,1% DTT (Dithiothreitol) and 1% Polyvinylpyrolidone.
Instrument	Flowcytometer: CyFlow Space (Partec GmbH, Otto Hahnstrasse 32, D-4400 Münster, Germany) with 50 mW, 532 nm green laser
Software	Flomax version 2.8 (Partec)
Cell population abundance	The copped solution, containing cell constituents and large tissue remnants, is passed through a nylon filter of 50 µm mesh size. After incubation of at least 30 minutes at room temperature, the filtered solution with stained nuclei is send through the flow cytometer CyFlow (Sysmex Partec GmbH). At least 3000 nuclei of the sample and the internal standard (Vinca major) were measured.
Gating strategy	No specific gating strategy was applied. The peaks of the nuclei were not disturbed by the noise signals.

- [ ] Tick this box to confirm that a figure exemplifying the gating strategy is provided in the Supplementary Information.
